# Modelling the complex nature of the tumor microenvironment: 3D tumor spheroids as an evolving tool

**DOI:** 10.1186/s12929-024-00997-9

**Published:** 2024-01-23

**Authors:** Daniel B. Rodrigues, Rui L. Reis, Rogério P. Pirraco

**Affiliations:** 1 3B’s Research Group, I3Bs, Research Institute on Biomaterials, Biodegradables and Biomimetics, University of Minho, Headquarters of the European Institute of Excellence On Tissue Engineering and Regenerative Medicine, AvePark, Parque de Ciência e Tecnologia, Zona Industrial da Gandra, Barco, 4805-017 Guimarães, Portugal; 2 ICVS/3B’s, PT Government Associate Laboratory, Braga, 4805-017 Guimarães, Portugal

**Keywords:** Spheroids, 3D tumor models, Cancer, Tumor microenvironment

## Abstract

Cancer remains a serious burden in society and while the pace in the development of novel and more effective therapeutics is increasing, testing platforms that faithfully mimic the tumor microenvironment are lacking. With a clear shift from animal models to more complex in vitro 3D systems, spheroids emerge as strong options in this regard. Years of development have allowed spheroid-based models to better reproduce the biomechanical cues that are observed in the tumor-associated extracellular matrix (ECM) and cellular interactions that occur in both a cell–cell and cell-ECM manner. Here, we summarize some of the key cellular interactions that drive tumor development, progression and invasion, and how successfully are these interactions recapitulated in 3D spheroid models currently in use in the field. We finish by speculating on future advancements in the field and on how these can shape the relevance of spherical 3D models for tumor modelling.

## Background

Over the past decade, increasing energy has been placed in the implementation of the 3 R’s principle in animal experimentation, especially regarding animal replacement. Several different efforts have pushed this movement forwards including motions from the European parliament 2021/2784 (RSP) which urge the acceleration of the transition to a world without the use of animals in research, regulatory testing and education. Additionally, several studies have reported that mouse models of human cancers present species-specific differences [1] such as size, life span, and organ morphology and physiology which ultimately may lead to erroneous interpretations of the efficacy of novels therapeutics during translation to human clinical application. In parallel, the growing knowledge on the role of the ECM on both physiological and pathological conditions like cancer, namely through the regulation of cell proliferation, differentiation, migration, survival and adhesion, has created the urge to adopt 3D culture platforms in detriment of non-biomimetic 2D tissue-culture plastics [2]. The effects of these 3D culture systems have shown to impact cell behavior at various levels ultimately affecting drug sensitivity through changes observed in cell morphology, cell viability and cell survival pathways among others [3].

All of this has contributed to the growing investment of the research community in building faithful cancer models that preserve the 3D architecture and the multicellular complexity of cancer tissue. Among those models are organotypic models (based on substrates like Matrigel^®^ or Collagen I), tumoroids, organ-on-a chip and 3D scaffold-based systems [4–7].

While many of these systems have been proven advantageous for different applications such as high-throughput screening of anti-tumor drugs or to study specific cell–cell/cell-ECM interactions in detail, increasing both their fidelity and biomimetic complexity is still a major need. Additionally, key features such as ECM biomechanical properties play a crucial role in these models as not only is ECM stiffness influencing cellular attachment [8] but also tumor progression [9] and therefore requires careful tuning. In order to add to the complexity of these disease models, the cell heterogeneity observed within the tumor microenvironment [10, 11] must be considered. Tumor heterogeneity has been a topic of interest in cancer research, with the first evidence of a heterotypic tumor model going back several decades [12]. The efforts to create highly complex tumor spheroid models which recreate the cellular interactions observed in the tumor microenvironment have been significant over the past years, with the use of non- cancerous cells (e.g. fibroblasts, endothelial cells, adipocytes, and immune cells) native to the tumor microenvironment key to this effort.

Spheroids emerged as 3D aggregates of cells that arrange themselves into sphere-like formations when in low-adhesion culture surfaces. These can be used to recapitulate tumor architecture when compared to conventional 2D cultures. Throughout this review, we will focus on how spheroid-based models have evolved, allowing for the development of tumor models with greater bio-similarity, how cellular interactions and heterogeneity have played a part in these advances and what can be expected for the future of these systems.

## The tumor microenvironment

In vitro tumor models have been contributing to the overall reduction of animal experimentation in the cancer field and to a more standardized use of models of higher complexity and biological relevance due to their high degree of reproducibility, high translational value and even commercial availability. However, several issues remain unresolved with a window of opportunity for new developments and advances. One of the main issues is the uniformity and reproducibility over a large number of samples, in which a uniform shape and size are difficult to attain. A second concern is defining a series of prerequisites that would qualify these systems as valid for drug screening. Ultimately, to take full advantage of these systems, high-throughput is a requirement, especially in the case of drug screening for pharmaceutical use.

**Fig. 1:**
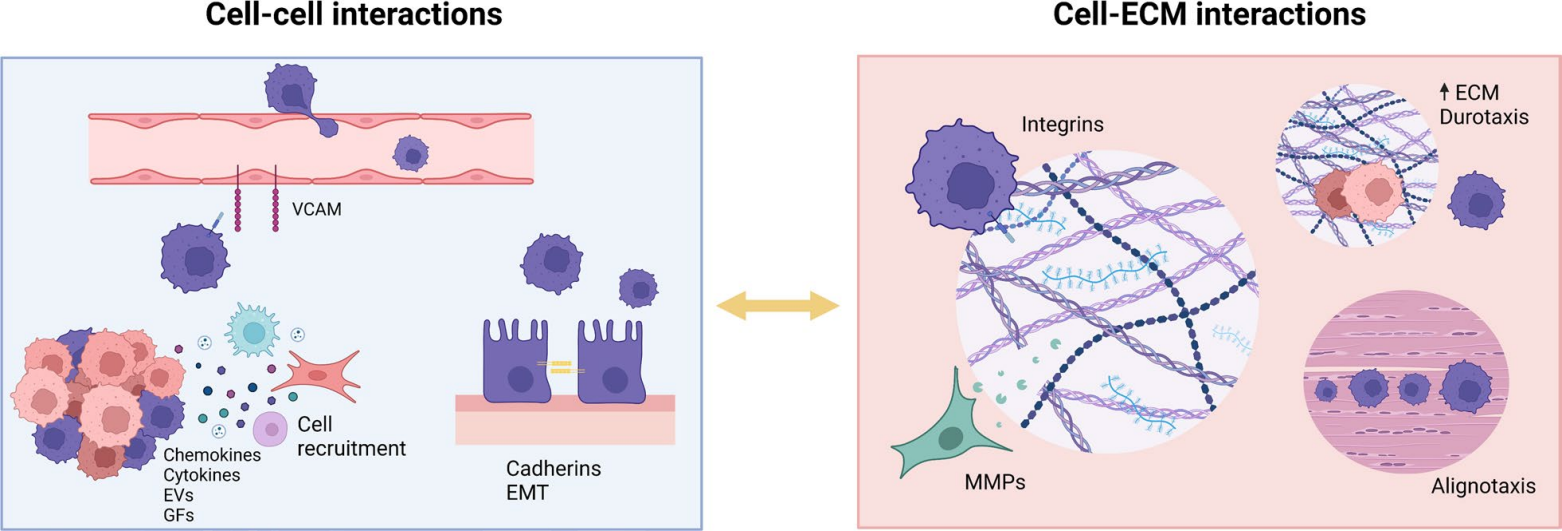
Cell interactions in the tumor microenvironment shape disease progression. The tumor microenvironment is a highly complex system that is tightly regulated by several different mechanisms. Most of these regulatory mechanisms occur in between cells, however interactions with the surrounding ECM are known to drive tumor cell migration and metastasization. A plethora of distinct biomolecules are behind these interactions which drive cell recruitment and chemotaxis, EMT, cell extravasation and cell invasion

To overcome these issues, a clearer understanding of the tumor microenvironment is required. Here, we will focus mainly on 2 areas of cellular processes critical in tumor biology: (1) cell–cell interactions and (2) cell-ECM interactions within the tumor microenvironment (Fig. 1). Attention will be given to key aspects driving tumor formation and progression, and how they may be applied during the development of novel 3D tumor models.

### Cell–cell interactions in the tumor microenvironment 

The cellular composition of a tumor makes for a heterogeneous architecture given its distinct cell types. Communication between these different cell types allows for the regulation of the microenvironment and therefore acts as one of the driving forces promoting tumor progression [13, 14]. However, to bridge the gap between native tissues and novel tumor models, it is necessary to first understand the interactions within the structural organization of current spheroid models. Variables such as time of culture and spheroid compactness contribute to how cell–cell interactions occur within these models, which in turn influences cancer cell behavior regarding proliferation, survival, and response to cancer therapeutics [15]. Within these systems, cell–cell cohesiveness is determined by key players such as cadherins or integrins [16–18] or intracellular components such as actin or microtubules [19, 20].

Thus, going forward, we will split cell–cell dynamics into two distinct approaches, cell–cell interactions through cell adhesion molecules (CAMs) and secondly cell–cell signaling mediated via biomolecules such as cytokines, chemokines, growth factors and exosomes.

CAMs are a multivalent family of cell surface proteins that are involved in several roles, such as intercellular, intracellular, and cell-extracellular matrix interactions, as well as cell growth and motility regulation, signal transduction pathways, and inflammation. These proteins are further subdivided into four distinct subfamilies: immunoglobulin-like adhesion molecules, integrins, cadherins, and selectins. Several of these molecules have been implicated in cancer formation and progression.

Selectins are cell surface lectins that mediate the adhesion of circulating cells to the endothelium. This class of adhesion molecules consists of three proteins: E (endothelial), L (leukocytes), and P (platelet)-selectin. The recruitment of leukocytes through the expression of these molecules within the tumor microenvironment has aided in immune invasion, dissemination, extravasation, and the formation of a metastatic niche [21–25]. Overexpression of selectin ligands by cancer cells has also contributed to poor patient prognosis [26, 27].

Similar to selectins, cadherins are responsible for cell–cell adhesion and are the most explored CAMs in the field of cancer. They are transmembrane glycoproteins involved in the maintenance of normal tissue architecture and have a particular role in organism growth. Cadherins can be further classified into several subtypes; however, E-cadherin and N-cadherin have been the most studied in the field of cancer. The expression of these classical cadherins has been associated with the epithelial-to-mesenchymal transition, a biological process in which polarized epithelial cells undergo a phenotypic switch into a mesenchymal cell phenotype [28]. Upon this switch, cells are prone to enhanced migratory capacity, invasiveness, greater resistance to apoptosis, and increased production of ECM components [28]. This is triggered by a decrease in the expression of the endothelial marker E-cadherin [29–31] followed by an increase in the expression of N-cadherin [32–34]. It is also important to highlight that this shift in phenotype may not be so black and white in which carcinoma cells may exhibit several epithelial-mesenchymal characteristics [35–37]. This mechanism is tightly regulated by a series of biomolecules, such as transforming growth factor-β (TGF-β), the fibroblast growth factor (FGF) family and epidermal growth factor (EGF) [38–45]. These soluble factors belong to a group of proteins known as growth factors which have been implicated in the constitutive activation of growth-promoting pathways, modulation of cell phenotype and promoting tumor neovascularization. Possibly one of the most studied is vascular endothelial growth factor (VEGF), a glycoprotein with an important role in endothelial cell proliferation and vascularization [46]. Within the tumor microenvironment, this molecule is produced by tumor associated macrophages as well as by cancer cells, contributing to the production of damaged, permeable and leaky neo-vessels [47, 48] that nevertheless support tumor survival [49]. Other growth factors such as Epidermal growth factor (EGF) and fibroblast growth factor (FGF) have been tied to cancer cell proliferation, differentiation and survival in the case of EGF [50–53], while FGF additionally acts as a pro-angiogenic factor by synergistically acting with VEGF [54, 55]. In a similar fashion, PDGF is a factor impacting cancer cell proliferation capabilities [56] that can also trigger the production of ECM proteins and support tumor angiogenesis [56, 57], a process in which the tumor microenvironment supports the formation of new blood vessels crucial for tumor cell can growth, invasion and metastasization. Another growth factor that has become a biomarker for tumor cell activity is insulin-like growth factor (IGF) [58, 59] which was also implicated in inhibiting tumor cell apoptosis while stimulating their proliferation [60, 61].

Several other soluble proteins have been associated with the direct regulation of the tumor microenvironment, including cytokines and chemokines. These are linked to regulating the nature of immune responses and controlling immune cell trafficking. Cytokines like tumor necrosis factor alpha (TNF-α), TGF-β, IL-6, IL-10 and IL-17 secreted in either an autocrine, endocrine or paracrine manner have assumed a pivotal role in promoting tumor survival and metastasis. This is achieved by several different pathways, may it be immune-suppression, inhibition of angiogenesis, vasculogenic mimicry and cancer cell proliferation, migration and invasion, or aggravating the inflammatory process [62–69]. Also crucial to the regulation of these cancer-related processes are chemokines. These are chemotactic cytokines that regulate the migration of immune cells and these have also been implicated in cancer processes [70]. Another form of soluble factor that has been tightly implicated in cancer are extracellular vesicles (EVs). EVs have gained quite some interest from the field due to their ability to transfer bioactive cargoes that have several effector functions. Linked to the transport of lipids, proteins, and nucleic acids, Evs have been implicated in the development and maintenance of tumor growth, metastasis and immune escape [71, 72].

### Cell-ECM interactions in the tumor microenvironment 

While cell–cell interactions are crucial in driving cancer processes, the role of ECM is also important. This non-cellular component found within all tissues and organs is not only responsible for the structural support of cellular constituents but also has a well-known role in establishing biochemical and biomechanical cues that are required for tissue morphogenesis, differentiation and homeostasis and that additionally have been involved in driving tumor progression as previously overviewed [73, 74]. Different key components contribute to the role of the ECM as a determinant factor for poor prognosis in several different cancers [73, 75]. Some of the most influential are metalloproteinases (MMPs) and integrins. MMPs are a family of zinc-containing endopeptidases, which are similar among themselves both structurally and functionally. These enzymes are known for their role tissue repair and remodeling, cellular differentiation, cell mobility and wound healing [76], achieved through the cleavage of ECM components [77]. Moreover, two gelatinases (MMP-2 and MMP-9) have caught the spotlight due to their implication in several mechanisms such as angiogenesis and infiltration of cancer cells as well as metastasization [78–80]. The production of these molecules has been associated to different non-malignant stromal cells such as fibroblasts immediately surrounding clusters of cancer cells [81, 82]. An imbalance in the production of MMP and its inhibitor TIMP has been identified as a key factor in driving poor prognosis [83, 84].

Changes in the structural nature of the ECM can, in turn, alter the mechanical properties of this non-cellular component and consequently lead to events such as durotaxis, in which cell migration occurs in response to gradients of extracellular stiffness [85, 86]. Similarly to durotaxis, cells have been reported to undergo directed migration along aligned ECM fibers defined as alignotaxis [87]. CAFs have been implicated in this process by promoting directional cancer cell migration through the alignment of fibronectin fibers within the tumor ECM [88].

Cells interact with ECM through a series of integrins which are a class of transmembrane αβ heterodimers that are responsible for the binding of extracellular matrix ligands, cell-surface ligands, and soluble factors [89]. Several domains have been identified, and at least 18 α and eight β subunits are known in humans [90, 91]. Different ligands such as the collagen, laminin, fibronectin or leukocyte-specific receptors, interact with these heterodimers in a distinct fashion. In this manner, integrin function embraces several cancer processes such as guiding tumor cell migration and invasion, cancer cell survival and anoikis suppression, extravasation and enhancement of tumor stemness. For instance, α3β1 is associated to the differentiation and maintenance of the CAF phenotype, while also supporting the invasion of pancreatic duct adenocarcinoma heterospheroids [92]. Other effects of integrins on fibroblasts have also been observed, with αbβ6 and α9β1 being implicated in CAF recruitment and in sustaining their survival [93, 94]. Integrin α9β1 has additionally been linked to promoting the migration of glioblastoma and osteosarcoma cells as well as metastatic progression [95]. In addition to cell surface expression of integrins, these have also been found in extracellular vesicles [96]. In a model of colorectal cancer, β1 integrin-rich EVs are secreted into circulation by tumor cells to activate resident fibroblasts in remote organs, which in turn induces a pre-metastatic niche and promotes metastatic cancer growth through the secretion of pro-inflammatory cytokines IL-6 and IL-8 [97]. Similarly, α2β1 aided in CAF EV uptake by lung fibroblasts and consequently the activation of the TGF-β signaling pathway in these cells [98].

## Modeling the tumor microenvironment in vitro

**Fig. 2:**
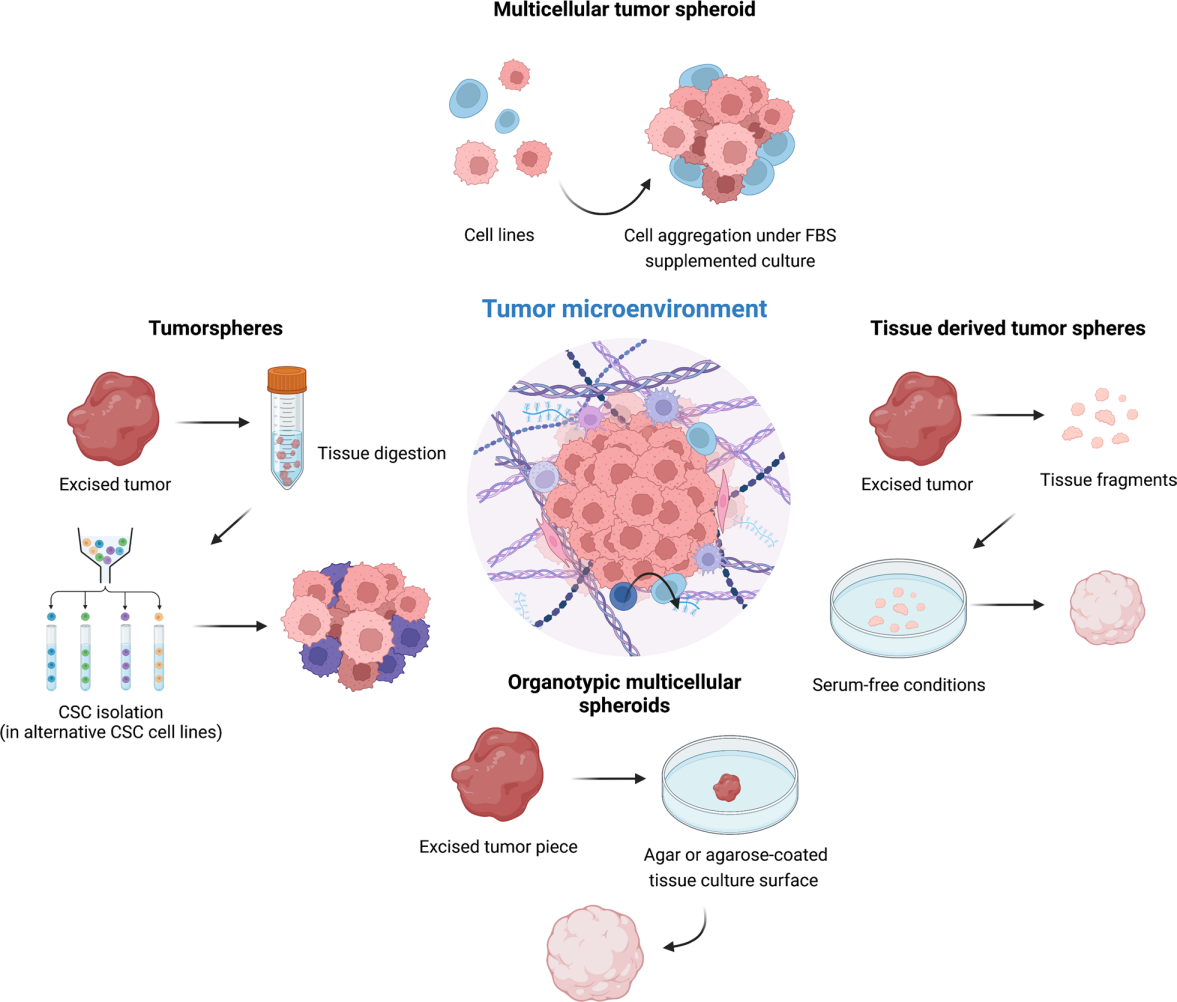
3D spheroid culture models for the in vitro recreation of the tumor microenvironment. Different culture methods have been adopted to attempt to recreate tissues within in vitro culture conditions. Spherical 3D models have been a golden standard in the field. Depending on cell-source, tissue digestion and culture methods a varying degree of complexity and homology towards native tumor tissues can be achieved

In order to develop 3D tumor models that are able to mimic interactions that take place within the tumor microenvironment, two key aspects come into play. The first is cell source and heterogeneity, which is an important differentiator for the different known spheroid tumor models, and the second is the level of recapitulation of key cell–cell and cell-ECM interactions that these models can achieve. Herein, we will briefly overview the different types of spheroid models that have already been described and the key features in terms of cellular interactions that have been recently studied (Fig. 2).

### Currently available spheroid models 

The nomenclature used for 3D tumor models has been quite heterogeneous over time, with several new terms being introduced every so often. Terms like “spheroids”, “sphere”, “tumorsphere”, “oncosphere”, “organoid” or “organotypic spheroid” have been deliberately used, often times making it difficult to understand the very nature of each system. These different models distinguish themselves mainly through medium composition used, culture surface, cell density, time required for formation, origin and handling.

There is however one common denominator that is the architecture of these systems. While the terms “aggregate” and “spheroids” have distinct meanings, confusion has been installed in using one term or the other. The systems that we will discuss herein are all “spheres” or “spheroids” consisting in closely compacted spherical cultures. And are not to be mistaken with “aggregates” that are loose cellular aggregates that easily detach [99]. Recently, Pasca et al., has overviewed the nomenclature of such systems in the context of nervous system models [100] while Weiswald et al. divided the classification of spherical cancer models into four main groups: (1) multicellular tumor spheroids, (2) tumorspheres, (3) tissue-derived tumor spheres, and (4) organotypic multicellular spheroids [101].

Here we will take a simplistic view to what has been used to name these systems in the context of cancer 3D models, based on the cell source and culture method (Table 1).

**Table 1:** Heterotypic spheroid-based tumor models

3D culture model	Culture technique	Strengths	Weaknesses	References
Multicellular tumor spheroid	- Use of ultra-low adhesion substrates (e.g. polystyrene culture plates)	-Metabolic and proliferative gradients similar in vivo -Clonality -Easy Maintenance -Ease of genetic manipulation	-Make use of FBS culture conditions -Originated from cell lines	[102, 108]
Tissue derived tumor sphere	-Partial digestion of cancer tissues into small fragments that form in spherical structures	-Serum-free culture -Native cell–cell contract is maintained -Maintenance of histological characteristics -Preservation of genetic phenotype and metastatic properties	-Deprived of stromal cells	[101, 110, 111]
Tumorsphere	-Mechanical and enzymatic dissociation of tumor samples into single cell suspensions	-Useful system to study CSC -Serum-free culture conditions	-Lack to fully recapitulate the TME -Require specific factors to favor stem cells growth	[113]
Organotypic multicellular spheroid	-Formed by excised tumors but without tissue digestion	-May be cryopreserved while maintaining their histological characteristics -Highly similar to native tumor tissues -High cellular heterogeneity -Presence of vascular, immune and stromal fractions	-Dependent on a low-adhesion substrate (e.g. agarose) for spheroid formation	[114, 115, 117]

Summary of spheroid-based tumor models including spheroid formation techniques, strengths and weaknesses

#### Multicellular tumor spheroids

The main discriminator between multicellular spheroids (MCS) and traditional 2D monolayers is that MCS are grown as spheres, which promotes enhanced cell–cell and cell-ECM adhesion. These cellular spheres are generated from single-cell suspension cultures in FBS supplemented medium without the supply of an exogenous ECM and generally originate from cancer cell lines and not from dissociated tissues. Whether cells can spontaneously aggregate into spheres in the absence of a cell attachment substrate is highly dependent on the cell type, therefore it is important to highlight that not all cell lines are capable of generating compact MCS [102]. However, it has been described that the interplay between long-chain ECM fibers with RGD motifs of dispersed cells and integrins on cell membranes are determinant for the initial steps of cell aggregation and spheroid formation [17, 103]. Studies have shown that in certain cell lines, an enhanced production of ECM constituents such as fibronectin and laminin are increased in 3D spheroid-based cultures when compared to 2D [104–107], which may explain why some cell lines not displaying this enhanced 3D ECM production are incapable of forming cohesive spheroids. While histological resemblance of these 3D culture systems with primary cancer tissues is minimal, they present metabolic and proliferative gradients seen in vivo as well as relevant chemoresistance. Several other key features make MCS desirable tumor models, such as their clonality, easy maintenance and simplicity in performing genetic manipulation. From a biological point of view, MCS can expand up to sizes between 1 to 3 mm in diameter. However, above 500 μm a distinct architecture is known to take over, comprising an outer proliferating layer followed internally by a layer of quiescent cells and housing in its center a necrotic core [101]. Growth dynamics also vary from traditional 2D cultures, where an early exponential phase is observed followed by a period of delayed growth resulting from the increase in the nonproliferating and necrotic cells [108].

Several methods have been proposed for the formation of MCS [109]; however, the most adopted among the community consists in providing conditions in which the adhesive forces between the cells are greater than between cells and the substrate on which they are cultured, as is the case of ultra-low adhesion tissue culture plates.

#### Tissue-derived tumor spheres

While MCS have aided in recreating cell–cell interactions specific to the tumor microenvironment in vitro, they are still limited in their complexity when taking into consideration the distinct cellular genotypes and phenotypes that make for heterogeneity between tumors of the same histopathological subtype. More biologically representative 3D spherical tumor models are tissue-derived tumor spheres (TDTSs) also called cancer tissue-originated spheroids. These systems are obtained from the partial digestion of cancer tissues into small fragments which then spontaneously form spherical structures within several hours under serum-free conditions [110]. One of the key advantages of these systems is that cell–cell contact is maintained throughout the preparation and culture process which yields spheroids consisting of highly purified and viable cancer cells [111]. This high yield in neoplastic cells may be explained by the strong cell–cell interactions observed between cancer cells, thus leading to the preservation of neoplastic cells during partial dissociation and the loss of non-neoplastic cells, resulting in TDTSs deprived of stromal cells [111]. Among their advantages as a biosimilar tumor model are their capacity to recapitulate avascular tumor regions as well as the maintenance of histological characteristics, gene expression profiles, mutations in relevant genes and tumorigenic and metastatic properties [101].

#### Tumorspheres

Cancer stem cells (CSCs) have become a hot topic due to their important role in tumor dissemination. The capacity for self-renewal and the ability to differentiate into other specialized cell types have been identified in certain subpopulations of cells within tumors, with the added features of being capable of seeding tumors when transplanted into an animal host [112]. Tumorspheres have been created not as another model to mimic cancer tissues but to study the properties of CSCs as it has been shown that tumorspheres do not fully replicate the 3D tumor structure nor environment [113]. Tumorspheres have been known to form when CSCs are plated at low density in nonadherent conditions which promote the proliferation of these cells as clonal nonadherent spherical clusters. These cultures are usually conducted in medium devoid of FBS and supplemented with several factors that favor stem cell growth. In order to first obtain these CSCs, the mechanical and enzymatic dissociation of tumor samples into single cell suspensions is required, but ultimately CSCs culture may also be obtained through cancer cells lines.

#### Organotypic multicellular spheroids (tumor tissues)

Organotypic Multicellular Spheroids (OMS) are very distinct from the classical MCS as they are generally obtained from excised tumor tissues similarly to TDTSs but without undergoing digestion. These tissue pieces are then transferred to agar or agarose-coated tissue culture surface in order for them to develop into multicellular spheroids [114, 115]. Also important to point out is the capacity of these models to be cryopreserved while retaining their histological characteristics and being subject to only minor phenotypic and genotypic changes after thawing [116]. This model sets itself apart from other tumor models because of its high similarity to native tumor tissues, achieved by avoiding any kind of dissociation process that may interfere with the tissue architecture and cellular organization. This in turn leads to a high cellular heterogeneity similar to that of the tumor by maintaining the presence of vascular, immune and stromal fractions [117] contributing to a comparable 3D model.

### Modelling cell–cell and cell-ECM interactions in current spheroid models of cancer

Modeling the aforementioned cell–cell and cell-ECM crosstalk in in vitro tumor models is a requirement for the field and therefore the creation of biomimetic models capable of modeling not only drug behavior but also those cellular interactions within the tumor microenvironment has become a priority. In similar fashion to what is described within the tumor microenvironment (Table 2), 3D tumor models must also present these cell interactions through CAMs and soluble factors while producing ECM that retains characteristics typically observed in tumor-associated ECM in vivo.

**Table 2:** Cell–cell and cell-ECM interactions within the tumor microenvironment

Interaction form	Mediators	Molecular changes	Changes in TME	References
**Cell–Cell**	Cadherins (e.g. E and N)	-Loss of cell polarity in epithelial cells together with cell–cell adhesions - Cadherin instability in tumors is facilitated by oncogenic exosomes which disrupt endothelial cell adhesion junctions by directly suppressing the expression of VE-cadherin	-Cadherin-switch defined by a loss of E-cadherin and an increase in the expression of N-cadherin during tumor progression, thus gaining migratory and invasive properties -Enhanced vascular permeability which facilitates metastasis	[198–202]
	Selectins (E, L and P-Selectins)	- Myeloid-derived suppressor cells (MDSCs) down-regulate L-selectin levels on naive T cells -Induction of E-selectin-dependent endothelial retractions and a subsequent modulation of tight junctions through dephosphorylation of VE-cadherin (loosening of endothelial VE-cadherin-based junctions) -Cancer cells expressing L-selectin can stick, roll and crawling on high endothelial venules -Elevated expression of P-selectin facilitates the adhesion of cancer cells to the endothelium -Platelets expressing S-selectin attach to cancer cells and release tumor-supporting factors (EGF, VEGF, FGF and TGF-β)	-Decreasing ability of naïve T cells to home to sites where they would be activated -Increase transendothelial migration of cancer cells through L-selectin-mediated interactions -Cancer cell migration to lymph nodes -Triggering of cancer associated thrombosis -Promotion of cancer metastasis through platelet-cancer interactions	[23, 203–205]
	Biomolecules (TGF-β, EGF, FGF, TNF-α, IL-6, IL-10 and IL-17)	-TGF- β and FGF upregulate the secretion of matrix proteases (MMP-2 and MMP-9) while down-regulating TIMP -GFs induce the loss of the epithelial E-cadherin and gain of the mesenchymal N-cadherin -EMT induced by TGF-β increased cell-surface levels of EGFR and prevents its physical interaction with E-cadherin -In response to several different growth factors (transforming growth factor (TGF)-β, hepatocyte growth factor (HGF), platelet-derived growth factor (PDGF) and fibroblast growth factor 2 (FGF-2)) healthy fibroblasts undergo a phenotypical shift -IL-1α exerts pro-angiogenic effects in cancer cells by activating JNK signaling and increasing VEGF expression - Cancer M2 macrophages secrete IL-4, IL-10, IL-19, IL-33, TGF-β, and epithelial growth factor (EGF) promoting tumor growth and metastasis -Mesenchymal cells and fibroblasts present in tumor tissues secrete FGF, VEGF, and CXCL-12 chemokine to promote growth, invasion, and metastasis of tumor cells	-Growth factors induce molecular switches of cellular adhesion -Enhancement of the invasive potential of cancer cells - Morphological shift from normal epithelial cells to nonmetastatic cancer cells -Transformation of tissue-resident fibroblasts or mesenchymal stem cells (MSCs) to cancer-associated fibroblasts -Regulation of tumor angiogenesis	[206–212]
**Cell-ECM**	Integrins MMPs	-Integrin clustering leads to the ability of cancer cells to bypass the requirement of ECM stiffness FAK activation therefore being able to grow on soft substrate -Integrin αvβ3 activates MMP2 specifically to facilitate cancer cell migration and invasion through ECM degradation -Integrin α9β1 overexpression in breast cancer promotes CAFs recruitment -Integrin αvβ3 together with vitronectin upregulate mTOR activity, overriding inhibition by hypoxia and facilitating tumor cell invasion -Hypoxia-induced MMP-13 overexpression translates into EMT and tumor invasion -Expression of alpha v beta 3 on cultured melanoma cells enabled their binding to MMP-2 in a proteolytically active form	- Extracellular cell matrix (ECM) degradation mediated by MMP loaded exosomes - Cell-mediated collagen degradation and motility, thereby promoting directed cellular invasion -Increased tumor matrix stiffness -Enhanced integrin signaling and proliferation	[213–220]

General view of key mediators responsible for cell–cell and cell-ECM interactions in tumors and changes that occur from these interactions

#### Intercellular adhesion

CAMs are not only crucial for the formation of spherical 3D models due to the requirement of cellular aggregation but are also one of the methods by which cells interact and communicate, which in the end becomes crucial for tumor development and invasion. Ultimately, the expression of some of these molecules may be used for targeting purposes for the delivery of novel therapeutics.

**Fig. 3:**
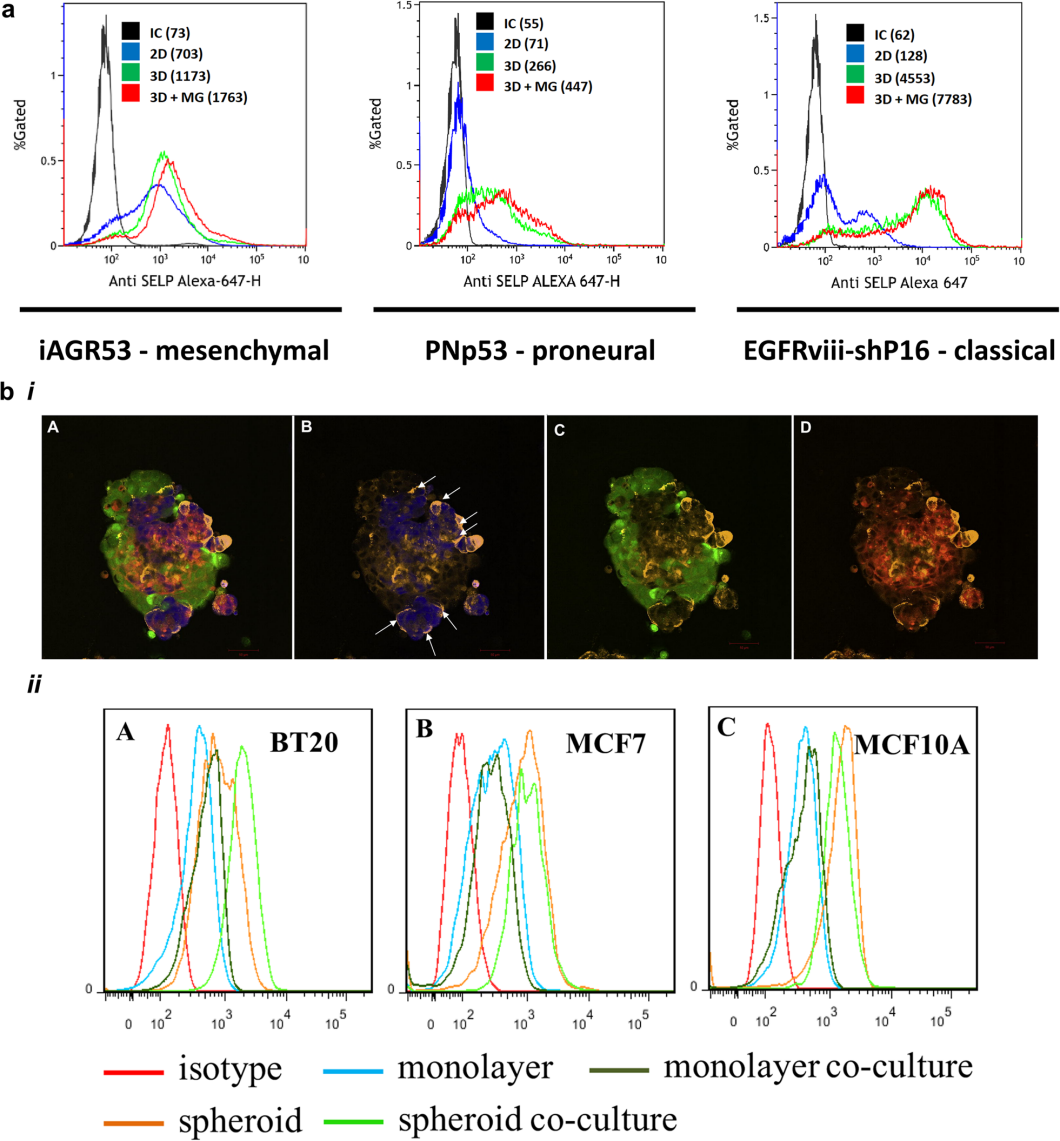
In vitro tumor spheroids as suitable tools to study cell adhesion molecules. **a** Given the high heterogeneity of GB tumors, P-selectin expression was studied in spheroids of mesenchymal (iAGR53 cell line), proneural (PNp53 cell line) and classical (EGFRviii-shP16 cell line) murine GB cell subtypes, when co-cultured in 3D with murine microglia and in their respective 2D controls. These distinct GB cell subtypes expressed high levels of P-selectin compared to 2D cultures and even higher levels when co-cultured with murine microglia. Adapted under the terms of CC 4.0 license from [121]. Copyright 2021, the authors. **bi** Confocal microscopy images of soluble E-selectin (orange) binding to BT20 (blue, B), MCF7 (green, C) or MCF10A (red, C) cells in whole co-culture spheroids (superimposed, A) on polydimethylsiloxane (PDMS) **bii** Flow cytometry histogram for soluble E-selectin binding in monolayer, spheroid, monolayer co-culture and spheroid co-culture with respect to isotype control for BT20 (A), MCF7 (B) and MCF10A (C) cells. Adapted with permission from [122]. Copyright 2012, Elsevier

P-selectin is a CAM with a proven role in tumor invasion [118–120]. Glioblastoma (GB) spheroids were studied to better understand how microglia could facilitate GB invasion and immunosuppression. It was found that P-selectin expression was higher in 3D cultures and in particular when microglia was co-cultured in GB spheroids in contrast to 2D cultures (Fig. 3a). This change in expression mediated the role of microglia in facilitating GB proliferation and invasion by altering the activation state of microglia/macrophages [121]. This confirms the importance of a 3D structure capable of mimicking the tumor microenvironment and associated ECM. Additionally, the expression of anti-inflammatory markers and cytokines IL-10 and TGF-β by microglia/macrophages were increased similarly to in vivo. Hematogenous metastasis is highly dependent on cell adhesion mediated by molecules like E-selectin expressed by the endothelial compartment of blood vessels. Understanding that such regulatory mechanisms also take place in 3D tumor models demonstrates their value in comparison to traditional culture systems. Homotypic and heterotypic 3D spheroids of tumorigenic (BT20 and MCF7) and non-tumorigenic (MCF10A) mammary cell lines have also been studied regarding their capability to bind E-selectin (Fig. 3bi) [122]. The authors claim that heterotypic 3D cultures demonstrated superior binding capacity of E-selectin to each cell type (Fig. 3bii) when compared to their respective 2D monolayer cultures together with a greater observed invasiveness, although this is not supported by the presented images. While the roles of E-selectin haven’t been deeply explored in complex 3D tumor spheroids comprising heterotypic cultures, other works have demonstrated the importance of this cell-adhesion molecule. Triculture tumor spheroids comprised of E-selectin expressing endothelial cells, normal human lung fibroblasts and human breast cancer cell line have been used to study an E-selectin drug delivery system for targeting tumor vasculature [123]. Tumor spheroids of Lewis lung carcinoma had been previously implanted in dorsal skinfold chambers of nude mice to study leukocyte adhesion in blood vessels induced by tumor spheroids. It was shown that blocking of E-selectin led to a slow rolling of leukocytes on the blood vessels [124]. In alternative systems such as flow-based assays, circulating cancer cells obtained from prostate cancer patients were shown to stably interact with E-selectin-expressing endothelial cells at physiological shear stress. Additionally, samples that were obtained during disease progression stages showed higher levels of interaction than those collected during times of therapeutic response [125].

Of late, several other groups have utilized 3D spherical culture systems to study interactions around cadherins and their implications in EMT and cancer cell invasion [126–129]. Cell–cell interactions through CAMs like N-cadherin and E-cadherin, ultimately impact cell contractility and hence cell dispersion or invasion. Heterotypic tumor spheroids consisting of EMT and epithelial A549 cancer cells, demonstrated these interactions through the formation of N-cadherin/E-cadherin adhesion complexes at the interface between highly contractile EMT cancer cells and poorly contractile epithelial cancer cells during tumor spheroid dispersion [130]. As a potential drug testing tool, multicellular spheroids of triple-negative breast cancer cells co-cultured with endothelial cells where prepared [131]. 3D culture led to an increase in the activation of the VE-cadherin pathway when endothelial cells were cultured in the presence of breast cancer cells, highlighting the importance of these models in recapitulating the tumor microenvironment in vitro.

#### Soluble factors

**Fig. 4:**
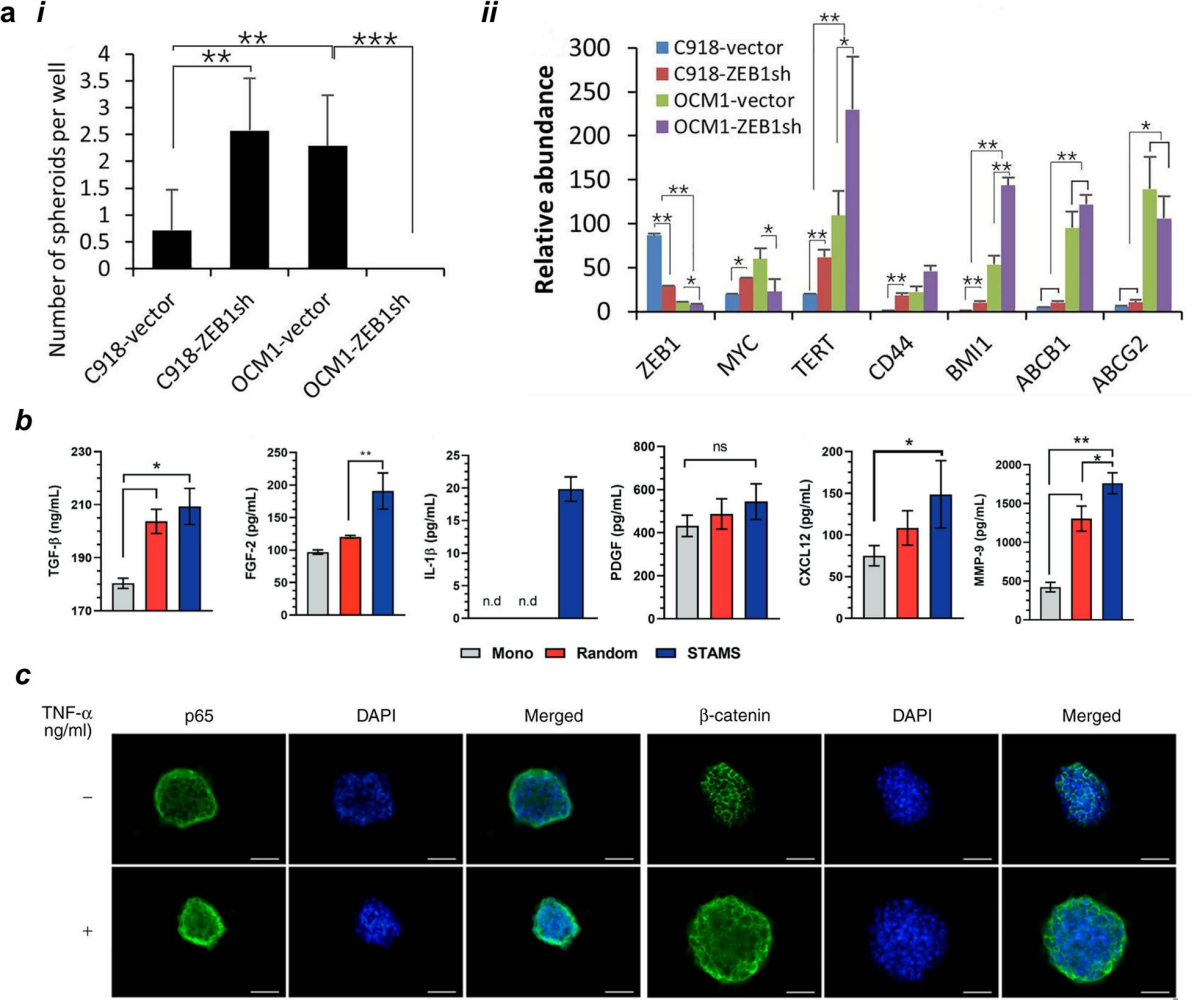
Soluble factors play a role in intercellular communication within 3D spheroid systems. **a*i*** ZEB1 knockdown in ICM1 (OCM-ZEB1sh) leads to diminished spheroid formation, suggesting the role of ZEB1 in spheroid formation. **a*ii*** ZEB1 directly binds to and potentially represses expression of the TERT and ABCB1 genes. Adapted under the terms of license CC BY-NC-ND 4.0 from [132]. Copyright 2022, the authors. **b** The secretion of EMT, chemoresistance and migratory–associated factors is heavily dependent on cellular organization in heterotypic tumor spheroids (Mono—Monotypic PANC-1 3D spheroid characterization; Random—Random PDAC microtumor 3D spheroids comprised by erratically distributed CAFs and PANC-1 cells (1:4 ratio); STAMS—PDAC tumor models assembled by cancer-associated fibroblasts addition to PANC-1 spheroids at day 14 of culture). Adapted with permission from [138]. Copyright 2021, Wiley. **c** TNF-α stimulation induces the activation of p65 and β-catenin in NCM460 cell spheroids when compared to non-stimulated spheroids. Adapted under the terms of license CC BY-NC-ND 4.0 from [162]. Copyright 2020, the authors

The cell relies on different mechanisms through which they inter-communicate, one of these key signaling mechanisms is through the release of soluble factors which should be similar in nature to in vivo tumors when developing spheroid tumor models. These soluble factors can play different roles in different pathways, one of which is the regulation of cell stemness. Given the importance of stem cell traits in cancer cells and their role in tumor development, spherical tumor models have been used to attempt to recreate the microenvironment in which these cells co-habit within the tumor. Recently, uveal melanoma (UM) cells, OCM and C918, were studied for their clonal heterogeneity in the form of non-adherent spheroid preparation [132]. OCM1 cells are representative of a low invasive potential and possess a spindle phenotype while C918 retain a higher invasive potential and are of the epithelioid phenotype. Considering this heterogeneity observed in UM, the authors aimed at understanding if there is evidence of a differential role of ZEB1 in different phenotypes of this disease. ZEB1 is a well-known transcription factor that plays a crucial part in tumor biology by driving cancer progression and metastization through the repression of E-Cadherin and consequent EMT promotion [133, 134]. Interestingly, the authors found that its expression was negatively correlated to spheroid formation from the single-cell suspension culture and to the expression of the stemness genes TERT, MYC, CD44, BMI1, ABCB1 and ABCG2, suggesting a possible role in the suppression of cancer stem cell properties in certain populations of UM (Fig. 4a, i-ii). While it would be expected, as in most carcinomas, that transcription factors such as the ones from SNAI, ZEB and TWIST families contributed to EMT and therefore to a phenotypic switch towards the mesenchymal and hence more aggressive phenotype, in UM the most aggressive form is the epithelioid (epithelial-like) phenotype [135]. Rather than EMT, MET has been observed in melanomagenesis and therefore the mechanisms that regulate tumor formation in UM are not the same as the ones observed in other carcinomas. To further add to the matter, in cutaneous melanomas, a molecular switch from ZEB2^high^/SNAI2^high^ to ZEB1^high^/TWIST1^high^ expression pattern has been associated to tumor initiation and progression together with increased aggressiveness [136, 137]. Therefore, when evaluating the role of ZEB1 in 3D tumor models one must consider its specific pathological mechanisms within a specific type of cancer.

While these systems can many times mimic specific cellular interactions that take place within the tumor microenvironment, they can also be created to achieve a higher resemblance with the desired cancer tissue architecture. A recent example of this was the creation of a pancreatic ductal adenocarcinoma model through the preparation of both multicellular spheroids and stratified multicellular spheroids that were produced by a 2-step process together with pancreatic CAF-Stellate Cells [138]. It was shown that this system presented stratification between cancer and stromal cells, accompanied by the expression of several soluble factors found in human pancreatic cancer such as TGF-β, FGF-2, IL-1β, and MMP-9 (Fig. 4b). Additionally, in this model, de novo deposition of collagen and glycosaminoglycans was observed. In a similar fashion, others have attempted to address the question of how soluble factors play a part in tumor development and progression [139–144].

Growth factors like IGF binding protein 3 (IGFBP3) have been studied given the role of the IGF-pathway in tumor cell proliferation, metastasis, and survival. For this purpose, H1299 cell lines transfected with IGFBP3 were used to produce 3D spheroids to study both growth and invasion [139]. It was shown that IGFBP3 negatively impacted 3D spheroids growth and invasion which correlated with an inhibition in the secretion of MMP-1 and an overall decrease in total MMP activity in culture supernatants. These results come in agreement to what has been reported regarding plasma IGFBP3 in lung cancer patients in which high expression was associated to lower tumor stages, while the number of metastatic sites correlated inversely with IGFBP3 plasma levels [139].

TGF has also been long recognized as important for the tumorigenic process, leading groups to develop new 3D culture systems in order to better understand how this cytokine interacts within the tumor microenvironment. Free floating spheroids were prepared from the human gastric cancer line MKN-45, which were then used to study the role of STAT3 activation [145]. These spheroid cultures presented higher STAT3 activity, up-regulation of TGF-β and VEGF with downregulation of IL-6. Additionally, conditioned medium from these gastric cancer spheroids were shown to polarize T cells towards a higher expression of FOXP3, TGF-b, and IL-10 indicative of a Treg phenotype. Another characteristic to consider while creating different 3D culture systems is geometry. While spheroids are valuable building blocks to mimic native tumor tissues in vitro, they many times lack geometrical complexity typical of tumors. To address this issue, researchers have studied the effects of different geometries in free standing tumor models and the impact of TGF-β signaling [140]. TGF-β appeared to upregulate the expression of cell tension-related proteins for peripheral cells, and alter the sensitivity of cells to their environment. Other recently reported systems have also observed the effects of TGF-β in tumor-simulated microenvironments recreated by 3D spherical models [146–150].

VEGF, a key player in the tumor microenvironment, has been immensely studied in vitro through spheroid cancer models. MG-63 human osteosarcoma cells were used to develop a 3D cancer model using microfluidics in order to study the role of VEGF-A in the tumor microenvironment [141]. A distinct behavior between 3 and 2D cell culture was observed, in which VEGF-A expression decreases upon the application of external stressors (reduced serum culture and HIF inhibition) in 2D cultures but increases while in 3D. Hepatocellular carcinoma, a highly vascularized tumor, was also modelled using 3D culture models for in vitro studies. For this effect, monocellular or co-culture spheroids were produced by seeding of either human liver hepatoma Huh7 (p53mut) and liver cancer HepG2 (p53^++^) or the co-culture of these at a 1:1 ratio with human umbilical vein endothelial cells (HUVECs) [126]. It was observed that VEGF stimulation led to changes in both size and density of the 3D spheroids, to an increase in invasion and angiogenesis as well as an increase in the expression of EMT markers vimentin, N-cadherin 2 and Thy-1. This importance of VEGF in the tumor process has also led to different heterotypic spheroid cultures comprising tumor cells and endothelial cells. For this effect, a 3D spheroid model of malignant pleural mesothelioma (MPM) was produced from either SPC111 cells obtained from biphasic MPM or P31 cells that are derived from epithelioid MPM [151, 152], representing the main cell types observed in MPM [153, 154]. P31 epithelioid MPM cells co-cultured with HUVEC cells permitted endothelial sprouting while SPC111 co-cultured spheroids repealed endothelial sprouts leading to anisotropic sprout arborization [152]. In another heterotypic spheroid model, long-term spheroids of up to 30 days of culture were developed from HCC1954 tumor cells, human fibroblasts, and ECs [155]. This study showed that EC were maintained viable for up to 1 month of culture under agitation while maintaining the expression of key surface markers and not requiring VEGF supplementation, as this growth factor was produced endogenously. Additionally, it was shown that this long-term maintenance is tumor cell line-dependent and, in some cases, dependent on the presence of fibroblasts and agitation. Additionally, several other GFs known to play a part in the cancer microenvironment have been studied in the context of 3D spheroid tumor models in vitro. Drug resistance mechanisms have been recently studied in several different spheroids of both melanoma and metastatic melanoma [142]. These studies revealed that the epidermal growth factor (EGF) pathway was affected by the triggering of protein kinase G, as indicated by diminished EGFR phosphorylation and decreased activation levels. Others have developed spheroids of human head and neck squamous cell carcinoma (HNSCC) cell lines with OECM-1 & SAS cells [156]. When stimulating these spheroids with EGF, changes to the surrounding collagen matrix were observed by means of a strong contraction deformation and a radial alignment of the collagen fibers with respect to the center of the spheroid. Additionally, growth factors such as fibroblast growth factor (FGF) [143, 157] and platelet-derived growth factor (PDGF) [144] have also been recently object of study in different 3D spheroid models underlying the importance of these models in mimicking the tumor microenvironment in vitro and the added value in using these systems when compared to traditional 2D cultures.

Beyond the role played by growth factors, other soluble factors such as cytokines and chemokines have been subject of study in the scope of these 3D spherical models. CXCCL12 is one of these molecules, a potent chemoattractant known to be involved in several different pathologies. A 3D co-culture model between triple negative 4T1 breast cancer cells and macrophages primed with either MSCCXCL12^+/+^ and MSCCXCL12^−/−^ was performed, in which it was observed that MSC-derived CXCL12 drove macrophages to support an increase in the number and size of 4T1 multicellular spheroids [158]. TNF expression in the cancer microenvironment has been modeled with several different forms of 3D spherical cultures. This cytokine has a strong involvement in cancer development with a mixed function as tumor suppressor or tumor promoter [159]. To understand the role of this cytokine on endothelial cells, spheroids of the human endothelial cell line Ea.hy926 were prepared and incubated with both TNF and VEGF. These 3D cultures resulted in increased the expression of both pro-inflammatory and anti-inflammatory factors when compared to the 2D condition [160]. In a tumor spheroid model of human gastric adenocarcinoma cell line HGC27, TNF-α and INF-γ were used as stimulants in order to trigger the production of PD-L1 which in turn allowed to study the mechanistic effects of PD-L1 blockade [161]. Zhao and colleges [162] aimed at understanding the effects of TNF-α in the malignant transformation of intestinal stem cells (Fig. 4c). TNF‑α proved to accelerate cell proliferation, migration and invasion, induce chemotherapy resistance and promote epithelial‑mesenchymal transition.

Finally, EVs have been an object of study by the research community given their potential as targets for novel therapeutics. Several works evaluate the expression of EVs in distinct spheroid models of cancer. In one study, CABA I human ovarian cancer cells, were cultured by a hanging drop method to create spheroids [163]. In these 3D cancer models, in similar fashion to in vivo tumors, EVs were secreted and entrapped inside the spheroid ECM. Additionally, these spheroids presented the capability of vasculogenic mimicry, defined by the formation of tubule-like structures in an angiongenesis-independent manner, capable of supporting the metabolic needs of growing tumors [164]. Over the past few years, several other groups presented different 3D culture systems able to express EVs in a similar fashion [165–167].

#### ECM

Tumor-associated ECM plays a major contribution to how cells organize and distribute themselves in the microenvironment and even in regulating their phenotype. Spheroids have been used to better understand some of the mechanisms underlying ECM interactions and unveil some of the pathways that can be targeted for future therapeutics.

As discussed before, integrins are crucial for the development of tumor metastasis. In epithelial ovarian cancer, the aberrant expression of integrins plays a crucial role in the detachment of malignant cells from the primary tumor and their reattachment to peritoneal surfaces. In a first step, the proteolytic activity of matrix metalloproteinases is required for the initial detachment of cancer cells from the primary tumor by cleaving α3-integrin on cancer cells [168]. This, together with the downregulation of E-cadherin resultant from EMT, leads to more invasive cancer cells [169], which in turn upregulates the expression of α5β1-integrin, thereby promoting the attachment of these cancer cells to secondary metastasis sites [170]. 3D spherical models of ovarian cancer have been used to shed light on the impact of both integrin beta-6 (ITGB6) and SET and MYN-domain containing 3 (SMYD3) on the activation of the TGFβ1/Smad3 pathway and downstream upregulation of N-cadherin and downregulation of E-cadherin [171]. This was indicative of the importance of the positive feedback loop between SMYD3/ITGB6/TGFβ1 in enhancing the invasion and adhesion of ovarian cancer spheroids as well as cell–cell communication in these models. Multicellular spheroids of ovarian carcinoma, prepared with the SKOV-3 cell line, have been used to better understand the role of Wnt11 on the expression of integrins and cadherin [172]. In these 3D models, Wnt11 was shown to negatively regulate ITGB2, ITGB6 and EpCAM while impeding the attachment of the multicellular spheroids to an ECM substrate, suggesting a role of this molecule in ovarian cancer progression. Other studies have also shown the importance of 3D models in studying the role of integrins in EMT. Thyroid cancer spheroids were used to study the effects of cadherin and integrins regarding motility and invasion in 3D [173]. Increased motility and a decrease in the molecular weight of integrin β1 were observed within the 3D model as well as an upregulation of EMT signaling molecules Snail and ILK, clearly denoting the differences between 3D and monolayer cultures. Tumor heterogeneity, as observed in melanoma, may result in poor treatment response. Therefore, studying tumor tissues in vitro may allow to unravel some of the key features that drive tumor aggressiveness. Tissue samples from melanoma patients were used to produce spheroids in order to better comprehend tumor aggressiveness [174]. On this basis, dermal nest melanoma cells displayed in vitro higher expression of α4/α7 integrin when compared to combined type melanoma cells. This difference among both was also visible in the expression of adhesion molecule N-cadherin, which was higher in dermal nest melanoma when compared to that of combined type melanoma. Hence, these models allowed to study the behavior of tissue derived cancer cells in specifically directed culture assays that allow to better understand the impact of certain molecular signatures and how they regulate tumor cell invasion.

**Fig. 5:**
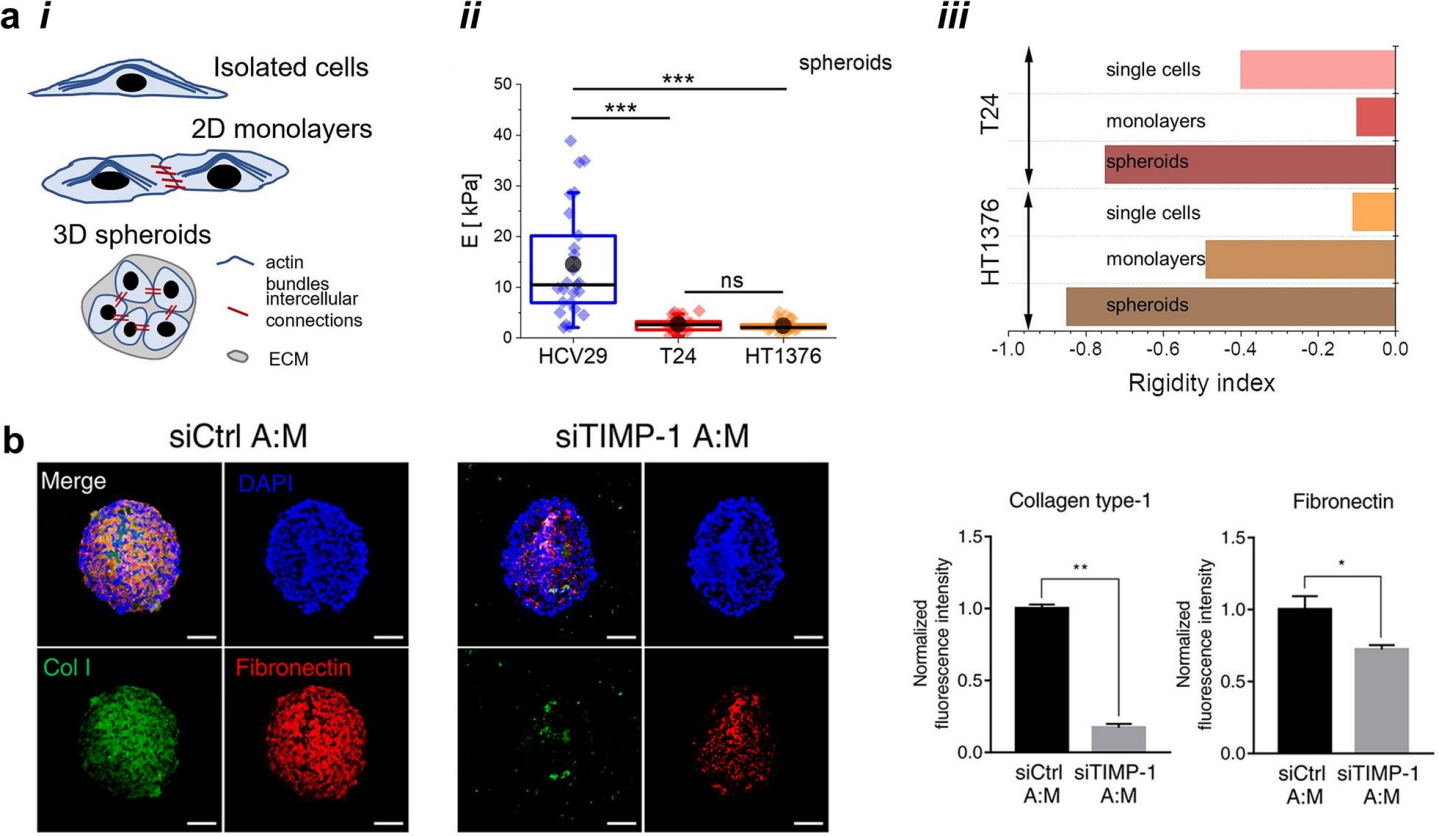
In vitro tumor spheroid models host tumor ECM mechanics. **a*i*** Actin cytoskeleton, intercellular connections, and ECM contributions to the overall mechanical properties at different culture conditions. **a*ii*** Analogous comparison between spheroids composed of normal and cancer cells. **a*iii*** Spheroids present the largest softening observed through the lowest rigidity index between different culture conditions, in relation to non-malignant HCV29 cells. Adapted under the terms of license CC BY 4.0 from [175]. Copyright 2022, the authors. **b** Effects of TIMP-1 silencing in hASC spheroids co-cultured with breast cancer cells on ECM expression. Collagen type I (green) and fibronectin (red) staining of tumor spheroids. Adapted under the terms of license CC BY 4.0 from [180]. Copyright 2022, the authors

Understanding microenvironment stiffness and mechanics, for instance, has become the objective of several recent reports that use tumor cell spheroids to perform mechanical analysis. Nanoindentation and microrheological experiments have been performed on spheroids of bladder cancer cell lines, which showed that cancer-induced changes lead to cell softening in T24 or HT1376 carcinoma cells when compared to HCV29 non-malignant ureter cancer cells (Fig. 5a, i-iii) [175]. Additionally, a decrease in the rigidity index was observed between cell monolayer and 3D cultures, indicating the role that ECM plays in microrheological properties. Other reports have equally described the use of different spheroid systems comprising multicellular or unicellular approaches to study the effects of mechanobiology on the tumor microenvironment [176–179]. Intertwined with ECM function are MMPs, which, as described above, have been associated with cancer cell invasion and are subject to various forms of regulation. The effect of intercellular communication and its impact on the production and activity of MMPs has been subject of study using 3D cancer models. Co-culture spheroids were produced using breast cancer cells (MDA-MB-231) and one of three different types of stromal cells, namely human adipose-derived stromal cells (hASCs), human bone marrow stromal cells or human dermal fibroblasts [180]. A greater deposition of both collagen type I and fibronectin was observed in the co-culture system with hASCs. This was found to be dependent on MMP activity, which, in turn, was regulated by increased expression of tissue inhibitor of metalloproteinases-1 (TIMP-1) (Fig. 5b) in the hASCs condition, consequently inhibiting ECM degradation. Furthermore, the presence of hASCs in the co-culture system led to a decrease in drug penetration efficacy, which may be a result of the higher deposition of surrounding ECM in this 3D tumor model. Understanding the role of MMPs and their respective inhibitors on the efficacy of drug penetration in benchtop 3D tumor models may open room for the translation of knowledge to the clinics and allow for the development of more effective therapeutics capable of hampering tumor progression in vivo.

The role of non-malignant tumor supporting cells within the tumor can also be addressed using 3D models. One such cell is the fibroblasts. The importance of fibroblasts within the tumor microenvironment has been evidenced in a 3D co-culture model encompassing HCT-8 colon cancer cells and NIH3T3 fibroblasts [181]. Herein, increased production of collagen type I and α-SMA was verified in the co-culture system when compared to monocellular spheroids. Increased stiffness was also observed as well as an increase in the abundance of TGF-β1. Additionally, this model allowed to observe the decrease in drug efficiency when going from 2D monoculture to 2D co-culture and to 3D cultures. Heterotypic models like the one described by Arora and colleagues [182], comprising lung adenocarcinoma cells (A549 and NCI-H460) combined with MRC-5 lung fibroblasts and THP-1 monocytes can be used to study these mechanisms. They showed that activated fibroblasts within the heterotypic spheroids massively expressed ECM component fibronectin and smooth muscle actin stress fibers. Furthermore, CD68 + pan-macrophages present in tumor spheroids at day 14 of culture underwent a possible myeloid lineage shift as observed by the increase in endothelial markers [182], which suggests a possible role of the enveloping tumor microenvironment in promoting this lineage conversion. While these CD68 + cells are typically known as tumor associated macrophages, studies have tied this shift in phenotype to what is called vascular mimicry [183], characterized by the formation of matrix-rich, vasculogenic-like channels completely independent of endothelial cells but displaying endothelial-like characteristics. Additionally highlighting the role of fibroblasts, a multicellular spheroid system was produced by the co-culture of dermal fibroblasts (NHDFs) and human primary mammary fibroblasts (HPMFs) with breast cancer-derived, tumorigenic cells (MDA-MB-231) [184]. Data showed that breast cancer cell line-conditioned medium activated both NHDFs and HPMFs, transitioning them into a CAF phenotype while contributing to a faster cellular aggregation observed by the release of MMPs. Transformed HPMFs additionally upregulated key molecules like fibroblast activation protein (FAP), vimentin, desmin, platelet-derived growth factor receptor A and S100 Calcium Binding Protein A4 (S100A4).

## The future of 3D spheroid cancer models

From the onset of tumor model development, using 2D monocellular cultures of cancer cells, many changes have occurred. The introduction of spheroids and their different variations has brought major developments to the field, allowing newer cancer models to become increasingly closer to mimicking the true tumor microenvironment. From monocellular tumor spheroids to more complex heterotypic patient-derived spheroids, the benefits of going 3D are convincing.

To better understand the benefits of these systems, recent studies have been performed to characterize their biomechanical similarity to native tumor ECM as well as the degree of recapitulation of native cell–cell and cell-ECM recapitulation. Establishing models that faithfully reproduce these interactions is even more important at a time where reducing the use of animal models while streamlining the testing of novel drugs and improving knowledge on key mechanisms driving tumor progression are a requirement. Growing emphasis will be placed on the necessity to unravel the role of cancer stem cells in the development of tumors and how they regulate cellular interactions within the microenvironment. Coupled to this is the heterogeneity observed within tumors, from different cell populations to cellular clonality; these features must be reproduced in newly developed 3D models. A good way to achieve this level of complexity will be through methods such as assembloids. Pașca et al. have described assembloids as self-organizing cellular systems resulting from the combination between different types of organoids possessing different specialized cell types, that result in integration. While first being generated by combining spheroids of human pluripotent stem cells driven to differentiate into region-specific organoids resembling either the dorsal pallium or the subpallium, and subsequently fused [185–188] they have recently been used to induce hepatic stem cell spheroids [189]. This methodology harbors several advantages over more traditional systems namely, the underlying possibility of maturing and tailoring individual organoids before fusion and replicating region-specific interfaces within tissues, as well as the longer viability time due to the more native-like representation of living systems [188, 190].

In order to additionally build the complexity of these tumor models, fusing different methodologies to simultaneously take advantage of their distinct features will also become routinely used. A form of achieving this is through the utilization of technologies like 3D printing and microfluidics or even in silico 3D modeling. Studies have already addressed the suitability of these fabrication technologies to develop 3D tumor analogues [191–197]. Additionally, the use of Artificial Intelligence at different stages of the development process will for sure bring for now unforeseeable benefits that cannot but leave a sense of excitement in the field.

## Conclusions

With the constant advances observed in the field over the past years, we expect tumor modelling to mature over the course of the next decade, supplying the global scientific community with improved tools allowing the developing of novel therapeutics that can ease the burden of cancer on society.

## Data Availability

Not applicable.

## References

[1] Cheon D-J, Orsulic S (2011). Mouse models of cancer. Annu Rev Pathol.

[2] Langhans SA (2018). Three-dimensional in vitro cell culture models in drug discovery and drug repositioning. Front Pharmacol.

[3] Breslin S, O’Driscoll L. The relevance of using 3D cell cultures, in addition to 2D monolayer cultures, when evaluating breast cancer drug sensitivity and resistance. Oncotarget; 2016;7(29). 10.18632/oncotarget.9935 PMC5216757 27304190

[4] Truong D, Puleo J, Llave A, Mouneimne G, Kamm RD, Nikkhah M (2016). Breast cancer cell invasion into a three dimensional tumor-stroma microenvironment. Sci Rep.

[5] Singh S, Ray LA, Shahi Thakuri P, Tran S, Konopka MC, Luker GD (2020). Organotypic breast tumor model elucidates dynamic remodeling of tumor microenvironment. Biomaterials.

[6] Rijal G, Li W (2022). A versatile 3D tissue matrix scaffold system for tumor modeling and drug screening. Sci Adv.

[7] Yokota E, Iwai M, Yukawa T, Yoshida M, Naomoto Y, Haisa M (2021). Clinical application of a lung cancer organoid (tumoroid) culture system. NPJ Precis Oncol.

[8] Lam CRI, Wong HK, Nai S, Chua CK, Tan NS, Tan LP (2014). A 3D biomimetic model of tissue stiffness interface for cancer drug testing. Mol Pharm.

[9] Najafi M, Farhood B, Mortezaee K (2019). Extracellular matrix (ECM) stiffness and degradation as cancer drivers. J Cell Biochem.

[10] Gerdes MJ, Sood A, Sevinsky C, Pris AD, Zavodszky MI, Ginty F. Emerging understanding of multiscale tumor heterogeneity. Front Oncol. 2014. 10.3389/fonc.2014.00366 PMC4270176 25566504

[11] Rich JN (2016). Cancer stem cells: understanding tumor hierarchy and heterogeneity. Medicine.

[12] Sutherland RM, McCredie JA, Inch WR (1971). Growth of multicell spheroids in tissue culture as a model of nodular carcinomas. J Natl Cancer Inst.

[13] Eugenin EA (2019). Role of cell-to-cell communication in cancer: new features, insights, and directions. Cancer Rep.

[14] Schwager SC, Taufalele PV, Reinhart-King CA (2019). Cell-cell mechanical communication in cancer. Cell Mol Bioeng.

[15] Cheng G, Tse J, Jain RK, Munn LL (2009). Micro-environmental mechanical stress controls tumor spheroid size and morphology by suppressing proliferation and inducing apoptosis in cancer cells. PLoS ONE.

[16] Ivascu A, Kubbies M (2007). Diversity of cell-mediated adhesions in breast cancer spheroids. Int J Oncol.

[17] Lin R-Z, Chou L-F, Chien C-CM, Chang H-Y (2006). Dynamic analysis of hepatoma spheroid formation: roles of E-cadherin and β1-integrin. Cell Tissue Res.

[18] Saias L, Gomes A, Cazales M, Ducommun B, Lobjois V (2015). Cell-cell adhesion and cytoskeleton tension oppose each other in regulating tumor cell aggregation. Cancer Res.

[19] Tzanakakis ES, Hansen LK, Hu W-S (2001). The role of actin filaments and microtubules in hepatocyte spheroid self-assembly. Cell Motil.

[20] Yoshii Y, Waki A, Yoshida K, Kakezuka A, Kobayashi M, Namiki H (2011). The use of nanoimprinted scaffolds as 3D culture models to facilitate spontaneous tumor cell migration and well-regulated spheroid formation. Biomaterials.

[21] Kang S-A, Blache CA, Bajana S, Hasan N, Kamal M, Morita Y (2016). The effect of soluble E-selectin on tumor progression and metastasis. BMC Cancer.

[22] Tremblay P-L, Auger FA, Huot J (2006). Regulation of transendothelial migration of colon cancer cells by E-selectin-mediated activation of p38 and ERK MAP kinases. Oncogene.

[23] Häuselmann I, Roblek M, Protsyuk D, Huck V, Knopfova L, Grässle S (2016). Monocyte induction of E-selectin-mediated endothelial activation releases VE-cadherin junctions to promote tumor cell extravasation in the metastasis cascade. Cancer Res.

[24] Ku AW, Muhitch JB, Powers CA, Diehl M, Kim M, Fisher DT, et al. Tumor-induced MDSC act via remote control to inhibit L-selectin-dependent adaptive immunity in lymph nodes. Elife. 2016;5. 10.7554/eLife.17375 PMC5199197 27929373

[25] Bal N, Kocer NE, Ertorer ME, Canpolat ET, Kayaselcuk F (2008). Maspin, E-selectin, and P-selectin expressions in papillary thyroid carcinomas and their correlation with prognostic parameters. Pathol Res Pract.

[26] Woodman N, Pinder ES, Tajadura V, Le Bourhis X, Gillett C, Delannoy P (2016). Two E-selectin ligands, BST-2 and LGALS3BP, predict metastasis and poor survival of ER-negative breast cancer. Int J Oncol.

[27] Tanio M, Muramoto A, Hoshino H, Murahashi M, Imamura Y, Yokoyama O (2021). Expression of functional E-selectin ligands on the plasma membrane of carcinoma cells correlates with poor prognosis in clear cell renal cell carcinoma. Urol Oncol Seminars Original Invest.

[28] Kalluri R, Neilson EG (2003). Epithelial-mesenchymal transition and its implications for fibrosis. J Clin Invest.

[29] Wendt MK, Taylor MA, Schiemann BJ, Schiemann WP (2011). Down-regulation of epithelial cadherin is required to initiate metastatic outgrowth of breast cancer. Mol Biol Cell.

[30] Aban CE, Lombardi A, Neiman G, Biani MC, La Greca A, Waisman A (2021). Downregulation of E-cadherin in pluripotent stem cells triggers partial EMT. Sci Rep.

[31] Burandt E, Lübbersmeyer F, Gorbokon N, Büscheck F, Luebke AM, Menz A (2021). E-Cadherin expression in human tumors: a tissue microarray study on 10,851 tumors. Biomark Res.

[32] Luo Y, Yu T, Zhang Q, Fu Q, Hu Y, Xiang M, et al. Upregulated N-cadherin expression is associated with poor prognosis in epithelial-derived solid tumours: a meta-analysis. Eur J Clin Invest. 2018;48. 10.1111/eci.12903 PMC5887888 29405291

[33] Mrozik KM, Blaschuk OW, Cheong CM, Zannettino ACW, Vandyke K (2018). N-cadherin in cancer metastasis, its emerging role in haematological malignancies and potential as a therapeutic target in cancer. BMC Cancer.

[34] Cao Z-Q, Wang Z, Leng P (2019). Aberrant N-cadherin expression in cancer. Biomed Pharmacother.

[35] Kröger C, Afeyan A, Mraz J, Eaton EN, Reinhardt F, Khodor YL (2019). Acquisition of a hybrid E/M state is essential for tumorigenicity of basal breast cancer cells. Proc Natl Acad Sci.

[36] Sinha D, Saha P, Samanta A, Bishayee A (2020). Emerging concepts of hybrid epithelial-to-mesenchymal transition in cancer progression. Biomolecules.

[37] Liao T-T, Yang M-H (2020). Hybrid epithelial/mesenchymal state in cancer metastasis: clinical significance and regulatory mechanisms. Cells.

[38] Yang B, Bai J, Shi R, Shao X, Yang Y, Jin Y (2020). TGFB2 serves as a link between epithelial-mesenchymal transition and tumor mutation burden in gastric cancer. Int Immunopharmacol.

[39] Basu M, Bhattacharya R, Ray U, Mukhopadhyay S, Chatterjee U, Roy SS (2015). Invasion of ovarian cancer cells is induced byPITX2-mediated activation of TGF-β and Activin-A. Mol Cancer.

[40] Kim BN, Ahn DH, Kang N, Yeo CD, Kim YK, Lee KY (2020). TGF-β induced EMT and stemness characteristics are associated with epigenetic regulation in lung cancer. Sci Rep.

[41] Hao Y, Xiao Y, Liao X, Tang S, Xie X, Liu R (2021). FGF8 induces epithelial-mesenchymal transition and promotes metastasis in oral squamous cell carcinoma. Int J Oral Sci.

[42] Adamczyk-Gruszka O, Horecka-Lewitowicz A, Gruszka J, Wawszczak-Kasza M, Strzelecka A, Lewitowicz P (2022). FGFR-2 and epithelial-mesenchymal transition in endometrial cancer. J Clin Med.

[43] Farajihaye Qazvini F, Samadi N, Saffari M, Emami-Razavi AN, Shirkoohi R (2019). Fibroblast growth factor-10 and epithelial-mesenchymal transition in colorectal cancer. EXCLI J.

[44] Xu Q, Zhang Q, Ishida Y, Hajjar S, Tang X, Shi H (2017). EGF induces epithelial-mesenchymal transition and cancer stem-like cell properties in human oral cancer cells via promoting Warburg effect. Oncotarget.

[45] Schelch K, Vogel L, Schneller A, Brankovic J, Mohr T, Mayer RL, et al. EGF induces migration independent of EMT or invasion in A549 lung adenocarcinoma cells . Front Cell Dev Biol. 2021. 10.3389/fcell.2021.634371 PMC7994520 33777943

[46] Ferrara N, Gerber H-P, LeCouter J (2003). The biology of VEGF and its receptors. Nat Med.

[47] Nagy JA, Feng D, Vasile E, Wong WH, Shih S-C, Dvorak AM (2006). Permeability properties of tumor surrogate blood vessels induced by VEGF-A. Lab Invest.

[48] Dvorak HF. Reconciling VEGF With VPF: the importance of increased vascular permeability for stroma formation in tumors, healing wounds, and chronic inflammation. Front Cell Dev Biol. 2021. 10.3389/fcell.2021.660609 PMC8021773 33834026

[49] Harmey JH, Bouchier-Hayes D (2002). Vascular endothelial growth factor (VEGF), a survival factor for tumour cells: Implications for anti-angiogenic therapy. BioEssays.

[50] Sasaki T, Hiroki K, Yamashita Y (2013). The role of epidermal growth factor receptor in cancer metastasis and microenvironment. Biomed Res Int.

[51] Tomas A, Futter CE, Eden ER (2014). EGF receptor trafficking: consequences for signaling and cancer. Trends Cell Biol.

[52] Wiedlocha A, Haugsten EM, Zakrzewska M (2021). Roles of the FGF-FGFR signaling system in cancer development and inflammation. Cells.

[53] Presta M, Chiodelli P, Giacomini A, Rusnati M, Ronca R (2017). Fibroblast growth factors (FGFs) in cancer: FGF traps as a new therapeutic approach. Pharmacol Ther.

[54] Cao R, Ji H, Feng N, Zhang Y, Yang X, Andersson P (2012). Collaborative interplay between FGF-2 and VEGF-C promotes lymphangiogenesis and metastasis. Proc Natl Acad Sci.

[55] Golfmann K, Meder L, Koker M, Volz C, Borchmann S, Tharun L (2018). Synergistic anti-angiogenic treatment effects by dual FGFR1 and VEGFR1 inhibition in FGFR1-amplified breast cancer. Oncogene.

[56] Farooqi AA, Siddik ZH (2015). Platelet-derived growth factor (PDGF) signalling in cancer: rapidly emerging signalling landscape. Cell Biochem Funct.

[57] Xue Y, Lim S, Yang Y, Wang Z, Jensen LDE, Hedlund E-M (2012). PDGF-BB modulates hematopoiesis and tumor angiogenesis by inducing erythropoietin production in stromal cells. Nat Med.

[58] Pohlman AW, Moudgalya H, Jordano L, Lobato GC, Gerard D, Liptay MJ (2022). The role of IGF-pathway biomarkers in determining risks, screening, and prognosis in lung cancer. Oncotarget.

[59] Ma C, Wang Y, Wilson KM, Mucci LA, Stampfer MJ, Pollak M (2022). Circulating insulin-like growth factor 1–related biomarkers and risk of lethal prostate cancer. JNCI Cancer Spectr..

[60] Su C, Wang W, Wang C (2018). IGF-1-induced MMP-11 expression promotes the proliferation and invasion of gastric cancer cells through the JAK1/STAT3 signaling pathway. Oncol Lett.

[61] Shanmugalingam T, Bosco C, Ridley AJ, Van Hemelrijck M (2016). Is there a role for IGF-1 in the development of second primary cancers?. Cancer Med.

[62] Sullivan KM, Jiang X, Guha P, Lausted C, Carter JA, Hsu C, et al. Blockade of interleukin 10 potentiates antitumour immune function in human colorectal cancer liver metastases. Gut. 2022;gutjnl-2021-325808. 10.1136/gutjnl-2021-325808 PMC9872249 35705369

[63] Itakura E, Huang R-R, Wen D-R, Paul E, Wünsch PH, Cochran AJ (2011). IL-10 expression by primary tumor cells correlates with melanoma progression from radial to vertical growth phase and development of metastatic competence. Mod Pathol.

[64] Mikkola T, Almahmoudi R, Salo T, Al-Samadi A (2022). Variable roles of interleukin-17F in different cancers. BMC Cancer.

[65] Zhang M, Zhang YY, Chen Y, Wang J, Wang Q, Lu H. TGF-β signaling and resistance to cancer therapy. Front Cell Dev Biol. 2021. 10.3389/fcell.2021.786728 PMC8669610 34917620

[66] van den Bulk J, de Miranda NFCC, ten Dijke P (2021). Therapeutic targeting of TGF-β in cancer: hacking a master switch of immune suppression. Clin Sci.

[67] Yoshimatsu Y, Wakabayashi I, Kimuro S, Takahashi N, Takahashi K, Kobayashi M (2020). TNF-α enhances TGF-β-induced endothelial-to-mesenchymal transition via TGF-β signal augmentation. Cancer Sci.

[68] Montfort A, Colacios C, Levade T, Andrieu-Abadie N, Meyer N, Ségui B. The TNF paradox in cancer progression and immunotherapy. Front Immunol. 2019. 10.3389/fimmu.2019.01818 PMC6685295 31417576

[69] Jones SA, Jenkins BJ (2018). Recent insights into targeting the IL-6 cytokine family in inflammatory diseases and cancer. Nat Rev Immunol.

[70] Ozga AJ, Chow MT, Luster AD (2021). Chemokines and the immune response to cancer. Immunity.

[71] Kugeratski FG, Santi A, Zanivan S (2022). Extracellular vesicles as central regulators of blood vessel function in cancer. Sci Signal..

[72] Lucotti S, Kenific CM, Zhang H, Lyden D (2022). Extracellular vesicles and particles impact the systemic landscape of cancer. EMBO J.

[73] Winkler J, Abisoye-Ogunniyan A, Metcalf KJ, Werb Z (2020). Concepts of extracellular matrix remodelling in tumour progression and metastasis. Nat Commun.

[74] Pickup MW, Mouw JK, Weaver VM (2014). The extracellular matrix modulates the hallmarks of cancer. EMBO Rep.

[75] Huang J, Zhang L, Wan D, Zhou L, Zheng S, Lin S (2021). Extracellular matrix and its therapeutic potential for cancer treatment. Signal Transduct Target Ther.

[76] Laronha H, Caldeira J (2020). Structure and function of human matrix metalloproteinases. Cells.

[77] Bode W, Maskos K (2003). Structural basis of the matrix metalloproteinases and their physiological inhibitors, the tissue inhibitors of metalloproteinases. Biol Chem.

[78] Fukushima R, Kasamatsu A, Nakashima D, Higo M, Fushimi K, Kasama H (2018). Overexpression of translocation associated membrane protein 2 leading to cancer-associated matrix metalloproteinase activation as a putative metastatic factor for human oral cancer. J Cancer.

[79] Miyake M, Goodison S, Lawton A, Gomes-Giacoia E, Rosser CJ (2015). Angiogenin promotes tumoral growth and angiogenesis by regulating matrix metallopeptidase-2 expression via the ERK1/2 pathway. Oncogene.

[80] Saito T, Kasamatsu A, Ogawara K, Miyamoto I, Saito K, Iyoda M (2015). Semaphorin7A promotion of tumoral growth and metastasis in human oral cancer by regulation of G1 cell cycle and matrix metalloproteases: possible contribution to tumoral angiogenesis. PLoS ONE.

[81] Polette M, Clavel C, Cockett M, Girod de Bentzmann S, Murphy G, Birembaut P (1993). Detection and localization of mRNAs encoding matrix metalloproteinases and their tissue inhibitor in human breast pathology. Invasion Metastasis.

[82] Ziani L, Safta-Saadoun TB, Gourbeix J, Cavalcanti A, Robert C, Favre G (2017). Melanoma-associated fibroblasts decrease tumor cell susceptibility to NK cell-mediated killing through matrix-metalloproteinases secretion. Oncotarget.

[83] Bourboulia D, Stetler-Stevenson WG (2010). Matrix metalloproteinases (MMPs) and tissue inhibitors of metalloproteinases (TIMPs): positive and negative regulators in tumor cell adhesion. Semin Cancer Biol.

[84] Wen X, Wu J-Q, Peng W, Feng J-F, Tang J-H (2015). MicroRNA-377 predicts poor clinical outcome of gastric cancer and induces tumorigenesis by targeting multiple tumor-suppressor genes. Oncol Rep.

[85] DuChez BJ, Doyle AD, Dimitriadis EK, Yamada KM (2019). Durotaxis by human cancer cells. Biophys J.

[86] Isomursu A, Park K-Y, Hou J, Cheng B, Shamsan G, Fuller B, et al. Negative durotaxis: cell movement toward softer environments. bioRxiv. 2020;2020.10.27.357178.

[87] Azimzade Y, Saberi AA, Sahimi M (2019). Regulation of migration of chemotactic tumor cells by the spatial distribution of collagen fiber orientation. Phys Rev E.

[88] Erdogan B, Ao M, White LM, Means AL, Brewer BM, Yang L (2017). Cancer-associated fibroblasts promote directional cancer cell migration by aligning fibronectin. J Cell Biol.

[89] Hynes RO (2002). Integrins: bidirectional, allosteric signaling machines. Cell.

[90] Hynes RO (1992). Integrins: versatility, modulation, and signaling in cell adhesion. Cell.

[91] Shimaoka M, Springer TA (2003). Therapeutic antagonists and conformational regulation of integrin function. Nat Rev Drug Discov.

[92] Cavaco ACM, Rezaei M, Caliandro MF, Lima AM, Stehling M, Dhayat SA (2018). The interaction between laminin-332 and α3β1 integrin determines differentiation and maintenance of CAFs, and supports invasion of pancreatic duct adenocarcinoma cells. Cancers (Basel)..

[93] Peng C, Zou X, Xia W, Gao H, Li Z, Liu N, et al. Integrin αvβ6 plays a bi-directional regulation role between colon cancer cells and cancer-associated fibroblasts. Biosci Rep. 2018;38. 10.1042/BSR20180243 PMC6435516 30355650

[94] Ota D, Kanayama M, Matsui Y, Ito K, Maeda N, Kutomi G (2014). Tumor-α9β1 integrin-mediated signaling induces breast cancer growth and lymphatic metastasis via the recruitment of cancer-associated fibroblasts. J Mol Med (Berl).

[95] Sun Z, Schwenzer A, Rupp T, Murdamoothoo D, Vegliante R, Lefebvre O (2018). Tenascin-C promotes tumor cell migration and metastasis through integrin α9β1-mediated YAP inhibition. Cancer Res.

[96] Soe ZY, Park EJ, Shimaoka M (2021). Integrin regulation in immunological and cancerous cells and exosomes. Int J Mol Sci.

[97] Ji Q, Zhou L, Sui H, Yang L, Wu X, Song Q (2020). Primary tumors release ITGBL1-rich extracellular vesicles to promote distal metastatic tumor growth through fibroblast-niche formation. Nat Commun.

[98] Kong J, Tian H, Zhang F, Zhang Z, Li J, Liu X (2019). Extracellular vesicles of carcinoma-associated fibroblasts creates a pre-metastatic niche in the lung through activating fibroblasts. Mol Cancer.

[99] Mayer B, Klement G, Kaneko M, Man S, Jothy S, Rak J (2001). Multicellular gastric cancer spheroids recapitulate growth pattern and differentiation phenotype of human gastric carcinomas. Gastroenterology.

[100] Pașca SP, Arlotta P, Bateup HS, Camp JG, Cappello S, Gage FH (2022). A nomenclature consensus for nervous system organoids and assembloids. Nature.

[101] Weiswald L-B, Bellet D, Dangles-Marie V (2015). Spherical cancer models in tumor biology. Neoplasia.

[102] Friedrich J, Seidel C, Ebner R, Kunz-Schughart LA (2009). Spheroid-based drug screen: considerations and practical approach. Nat Protoc.

[103] Cui X, Hartanto Y, Zhang H (2017). Advances in multicellular spheroids formation. J R Soc Interface.

[104] Santini MT, Rainaldi G, Indovina PL (2000). Apoptosis, cell adhesion and the extracellular matrix in the three-dimensional growth of multicellular tumor spheroids. Crit Rev Oncol Hematol.

[105] Glimelius B, Norling B, Nederman T, Carlsson J (1988). Extracellular matrices in multicellular spheroids of human glioma origin: increased incorporation of proteoglycans and fibronectin as compared to monolayer cultures. APMIS.

[106] Nederman T, Norling B, Glimelius B, Carlsson J, Brunk U (1984). Demonstration of an extracellular matrix in multicellular tumor spheroids. Cancer Res.

[107] Kelm JM, Timmins NE, Brown CJ, Fussenegger M, Nielsen LK (2003). Method for generation of homogeneous multicellular tumor spheroids applicable to a wide variety of cell types. Biotechnol Bioeng.

[108] Sutherland RM (1979). Cell and environment interactions in tumor microregions: the multicell spheroid model. Science.

[109] Lazzari G, Couvreur P, Mura S (2017). Multicellular tumor spheroids: a relevant 3D model for the in vitro preclinical investigation of polymer nanomedicines. Polym Chem.

[110] Yoshida T, Okuyama H, Endo H, Inoue M (2018). Spheroid cultures of primary urothelial cancer cells: cancer tissue-originated spheroid (CTOS) method. Methods Mol Biol.

[111] Kondo J, Endo H, Okuyama H, Ishikawa O, Iishi H, Tsujii M (2011). Retaining cell–cell contact enables preparation and culture of spheroids composed of pure primary cancer cells from colorectal cancer. Proc Natl Acad Sci.

[112] Yu Z, Pestell TG, Lisanti MP, Pestell RG (2012). Cancer stem cells. Int J Biochem Cell Biol.

[113] Valent P, Bonnet D, De Maria R, Lapidot T, Copland M, Melo JV (2012). Cancer stem cell definitions and terminology: the devil is in the details. Nat Rev Cancer.

[114] Rajcevic U, Knol JC, Piersma S, Bougnaud S, Fack F, Sundlisaeter E (2014). Colorectal cancer derived organotypic spheroids maintain essential tissue characteristics but adapt their metabolism in culture. Proteome Sci.

[115] Kaaijk P, Troost D, Das PK, Leenstra S, Bosch DA (1995). Long-term culture of organotypic multicellular glioma spheroids: a good culture model for studying gliomas. Neuropathol Appl Neurobiol.

[116] Sundlisaeter E, Wang J, Sakariassen PØ, Marie M, Mathisen JR, Karlsen BO (2006). Primary glioma spheroids maintain tumourogenicity and essential phenotypic traits after cryopreservation. Neuropathol Appl Neurobiol.

[117] Bjerkvig R, Tønnesen A, Laerum OD, Backlund EO (1990). Multicellular tumor spheroids from human gliomas maintained in organ culture. J Neurosurg.

[118] Mannori G, Crottet P, Cecconi O, Hanasaki K, Aruffo A, Nelson RM (1995). Differential colon cancer cell adhesion to E-, P-, and L-selectin: role of mucintype glycoproteins1. Cancer Res.

[119] Gong L, Mi H-J, Zhu H, Zhou X, Yang H (2012). P-selectin-mediated platelet activation promotes adhesion of non-small cell lung carcinoma cells on vascular endothelial cells under flow. Mol Med Rep.

[120] Ludwig RJ, Boehme B, Podda M, Henschler R, Jager E, Tandi C (2004). Endothelial P-selectin as a target of heparin action in experimental melanoma lung metastasis. Cancer Res.

[121] Yeini E, Ofek P, Pozzi S, Albeck N, Ben-Shushan D, Tiram G (2021). P-selectin axis plays a key role in microglia immunophenotype and glioblastoma progression. Nat Commun.

[122] Chandrasekaran S, Geng Y, DeLouise LA, King MR (2012). Effect of homotypic and heterotypic interaction in 3D on the E-selectin mediated adhesive properties of breast cancer cell lines. Biomaterials.

[123] Chantarasrivong C, Okada R, Yamane Y, Yang X, Higuchi Y, Konishi M (2022). Disposition of E-selectin-targeting liposomes in tumor spheroids with a perfusable vascular network. Drug Metab Pharmacokinet.

[124] Borgström P, Hughes GK, Hansell P, Wolitsky BA, Sriramarao P (1997). Leukocyte adhesion in angiogenic blood vessels. Role of E-selectin, P-selectin, and beta2 integrin in lymphotoxin-mediated leukocyte recruitment in tumor microvessels. J Clin Invest.

[125] Gakhar G, Navarro VN, Jurish M, Lee GY, Tagawa ST, Akhtar NH (2013). Circulating tumor cells from prostate cancer patients interact with e-selectin under physiologic blood flow. PLoS ONE.

[126] Rawal P, Tripathi DM, Nain V, Kaur S (2022). VEGF-mediated tumour growth and EMT in 2D and 3D cell culture models of hepatocellular carcinoma. Oncol Lett.

[127] Teveroni E, Di Nicuolo F, Vergani E, Bianchetti G, Bruno C, Maulucci G (2022). PTTG1/ZEB1 axis regulates E-cadherin expression in human seminoma. Cancers (Basel)..

[128] Kalender M, Bulbul MV, Kolbasi B, Keskin I (2022). In 2D and 3D cell culture models, effects of endothelial cells on E-cadherin / β-catenin expression levels and spheroid sizes in Ishikawa cells. Asian Pac J Cancer Prev.

[129] Perrin L, Belova E, Bayarmagnai B, Tüzel E, Gligorijevic B (2022). Invadopodia enable cooperative invasion and metastasis of breast cancer cells. Commun Biol.

[130] Zhang Q, Lin F, Huang J, Xiong C (2022). Mechanical transmission enables EMT cancer cells to drive epithelial cancer cell migration to guide tumor spheroid disaggregation. Sci China Life Sci.

[131] Dembélé P, Garnier O, Martin DK, Vilgrain I (2022). Microtumor spheroids provide a model for studying molecules involved in vascular organization: an illustrative study for VE-cadherin. Anticancer Res.

[132] Chen Y, Lu X, Gao L, Dean DC, Liu Y (2022). Spheroid-induced heterogeneity and plasticity of uveal melanoma cells. Cell Oncol (Dordr).

[133] Liu Y, El-Naggar S, Darling DS, Higashi Y, Dean DC (2008). Zeb1 links epithelial-mesenchymal transition and cellular senescence. Development.

[134] Zhang P, Sun Y, Ma L (2015). ZEB1: at the crossroads of epithelial-mesenchymal transition, metastasis and therapy resistance. Cell Cycle.

[135] Augsburger JJ, Gamel JW (1990). Clinical prognostic factors in patients with posterior uveal malignant melanoma. Cancer.

[136] Caramel J, Papadogeorgakis E, Hill L, Browne GJ, Richard G, Wierinckx A (2013). A switch in the expression of embryonic EMT-inducers drives the development of malignant melanoma. Cancer Cell.

[137] Chen Y, Lu X, Montoya-Durango DE, Liu Y-H, Dean KC, Darling DS (2017). ZEB1 regulates multiple oncogenic components involved in uveal melanoma progression. Sci Rep.

[138] Monteiro MV, Gaspar VM, Mendes L, Duarte IF, Mano JF (2021). Stratified 3D microtumors as organotypic testing platforms for screening pancreatic cancer therapies. Small Methods.

[139] Kuhn H, Frille A, Petersen MA, Oberhuber-Kurth J, Hofmann L, Gläser A (2022). IGFBP3 inhibits tumor growth and invasion of lung cancer cells and is associated with improved survival in lung cancer patients. Transl Oncol.

[140] Xie J, Hu X, Chen L, Piruska A, Zheng Z, Bao M (2022). The effect of geometry and TGF-β signaling on tumor cell migration from free-standing microtissues. Adv Healthc Mater.

[141] Sarkar S, Peng C-C, Tung Y-C (2020). Comparison of VEGF-A secretion from tumor cells under cellular stresses in conventional monolayer culture and microfluidic three-dimensional spheroid models. PLoS ONE.

[142] Quadri M, Comitato A, Palazzo E, Tiso N, Rentsch A, Pellacani G (2022). Activation of cGMP-dependent protein kinase restricts melanoma growth and invasion by interfering with the EGF/EGFR pathway. J Investig Dermatol.

[143] Otte J, Dizdar L, Behrens B, Goering W, Knoefel WT, Wruck W (2019). FGF signalling in the self-renewal of colon cancer organoids. Sci Rep.

[144] Goldsmith ZK, Coppess W, Irvine AS, Yuan K, Barsh SR, Ritter MK (2018). Targeting the platelet-derived growth factor-beta stimulatory circuitry to control retinoblastoma seeds. Invest Ophthalmol Vis Sci.

[145] Hajimoradi M, Rezalotfi A, Esmaeilnejad-Ahranjani P, Mohammad Hassan Z, Ebrahimi M (2022). STAT3 inactivation suppresses stemness properties in gastric cancer stem cells and promotes Th17 in Treg/Th17 balance. Int Immunopharmacol.

[146] Lee S-Y, Teng Y, Son M, Ku B, Hwang HJ, Tergaonkar V (2021). Three-dimensional aggregated spheroid model of hepatocellular carcinoma using a 96-pillar/well plate. Molecules.

[147] Puthdee N, Sriswasdi S, Pisitkun T, Ratanasirintrawoot S, Israsena N, Tangkijvanich P (2022). The LIN28B/TGF-β/TGFBI feedback loop promotes cell migration and tumour initiation potential in cholangiocarcinoma. Cancer Gene Ther.

[148] Djediai S, Gonzalez Suarez N, El Cheikh-Hussein L, Rodriguez Torres S, Gresseau L, Dhayne S (2021). MT1-MMP cooperates with TGF-β receptor-mediated signaling to trigger SNAIL and induce epithelial-to-mesenchymal-like transition in U87 glioblastoma cells. Int J Mol Sci.

[149] Fujishita T, Kojima Y, Kajino-Sakamoto R, Mishiro-Sato E, Shimizu Y, Hosoda W, et al. The cAMP/PKA/CREB and TGF-β/SMAD4 pathways regulate stemness and metastatic potential in colorectal cancer cells. Cancer Res. 2022;CAN-22-1369. 10.1158/0008-5472.CAN-22-1369 36066360

[150] SenGupta S, Hein LE, Xu Y, Zhang J, Konwerski JR, Li Y (2021). Triple-negative breast cancer cells recruit neutrophils by secreting TGF-β and CXCR2 ligands. Front Immunol.

[151] Hoda MA, Pirker C, Dong Y, Schelch K, Heffeter P, Kryeziu K (2016). Trabectedin is active against malignant pleural mesothelioma cell and xenograft models and synergizes with chemotherapy and Bcl-2 inhibition in vitro. Mol Cancer Ther.

[152] Kovacs I, Bugyik E, Dezso K, Tarnoki-Zach J, Mehes E, Gulyas M (2022). Malignant pleural mesothelioma nodules remodel their surroundings to vascularize and grow. Transl Lung Cancer Res.

[153] Jaurand M-C, Fleury-Feith J (2005). Pathogenesis of malignant pleural mesothelioma. Respirology.

[154] Beasley MB, Galateau-Salle F, Dacic S. Pleural mesothelioma classification update. 10.1007/s00428-021-03031-7. 10.1007/s00428-021-03031-7 33475835

[155] Franchi-Mendes T, Lopes N, Brito C (2021). Heterotypic tumor spheroids in agitation-based cultures: a scaffold-free cell model that sustains long-term survival of endothelial cells. Front Bioeng Biotechnol.

[156] Chen Y-Q, Kuo J-C, Wei M-T, Chen Y-C, Yang M-H, Chiou A (2019). Early stage mechanical remodeling of collagen surrounding head and neck squamous cell carcinoma spheroids correlates strongly with their invasion capability. Acta Biomater.

[157] De Bacco F, Orzan F, Erriquez J, Casanova E, Barault L, Albano R (2021). ERBB3 overexpression due to miR-205 inactivation confers sensitivity to FGF, metabolic activation, and liability to ERBB3 targeting in glioblastoma. Cell Rep.

[158] Babazadeh S, Nassiri SM, Siavashi V, Sahlabadi M, Hajinasrollah M, Zamani-Ahmadmahmudi M (2021). Macrophage polarization by MSC-derived CXCL12 determines tumor growth. Cell Mol Biol Lett.

[159] Sheng Y, Li F, Qin Z. TNF Receptor 2 makes tumor necrosis factor a friend of tumors. Front Immunol. 2018. 10.3389/fimmu.2018.01170 PMC5985372 29892300

[160] Gapizov SS, Petrovskaya LE, Shingarova LN, Svirschevskaya EV, Dolgikh DA, Kirpichnikov MP (2018). The effect of TNF and VEGF on the properties of Eahy926 endothelial cells in a model of multi-cellular spheroids. Acta Naturae..

[161] Zhou S, Zhu M, Meng F, Shao J, Xu Q, Wei J (2019). Evaluation of PD-1 blockade using a multicellular tumor spheroid model. Am J Transl Res.

[162] Zhao X, Ma L, Dai L, Zuo D, Li X, Zhu H (2020). TNF-α promotes the malignant transformation of intestinal stem cells through the NF-κB and Wnt/β-catenin signaling pathways. Oncol Rep.

[163] Giusti I, Poppa G, D’Ascenzo S, Esposito L, Vitale AR, Calvisi G (2022). Cancer three-dimensional spheroids mimic in vivo tumor features, displaying “inner” extracellular vesicles and vasculogenic mimicry. Int J Mol Sci.

[164] Angara K, Borin TF, Arbab AS (2017). Vascular mimicry: a novel neovascularization mechanism driving anti-angiogenic therapy (AAT) resistance in glioblastoma. Transl Oncol.

[165] Choi SA, Koh EJ, Kim RN, Byun JW, Phi JH, Yang J (2020). Extracellular vesicle-associated miR-135b and -135a regulate stemness in Group 4 medulloblastoma cells by targeting angiomotin-like 2. Cancer Cell Int.

[166] Bordanaba-Florit G, Madarieta I, Olalde B, Falcón-Pérez JM, Royo F (2021). 3D cell cultures as prospective models to study extracellular vesicles in cancer. Cancers (Basel)..

[167] Sadovska L, Zandberga E, Sagini K, Jēkabsons K, Riekstiņa U, Kalniņa Z (2018). A novel 3D heterotypic spheroid model for studying extracellular vesicle-mediated tumour and immune cell communication. Biochem Biophys Res Commun.

[168] Moss NM, Barbolina MV, Liu Y, Sun L, Munshi HG, Stack MS (2009). Ovarian cancer cell detachment and multicellular aggregate formation are regulated by MT1-MMP: a potential role in intra-peritoneal metastatic dissemination. Cancer Res.

[169] Veatch AL, Carson LF, Ramakrishnan S (1994). Differential expression of the cell-cell adhesion molecule E-cadherin in ascites and solid human ovarian tumor cells. Int J Cancer.

[170] Sawada K, Mitra AK, Radjabi AR, Bhaskar V, Kistner EO, Tretiakova M (2008). Loss of E-cadherin promotes ovarian cancer metastasis via α5-integrin, which is a therapeutic target. Cancer Res.

[171] Jiang Y, Zhou T, Shi Y, Feng W, Lyu T. A SMYD3/ITGB6/TGFβ1 positive feedback loop promotes the invasion and adhesion of ovarian cancer spheroids. Front Oncol. 2021. 10.3389/fonc.2021.690618 PMC8490739 34621667

[172] Azimian-Zavareh V, Hossein G, Ebrahimi M, Dehghani-Ghobadi Z (2018). Wnt11 alters integrin and cadherin expression by ovarian cancer spheroids and inhibits tumorigenesis and metastasis. Exp Cell Res.

[173] Kim B, Im N, Yang Daniel T, Kim J, Jung K, Kim Hoon T (2020). Enhancement of aberrantly modified integrin-mediated cell motility in multicellular tumor spheroids. Int J Oncol.

[174] Marconi A, Quadri M, Farnetani F, Ciardo S, Palazzo E, Lotti R (2022). In vivo melanoma cell morphology reflects molecular signature and tumor aggressiveness. J Investig Dermatol.

[175] Gnanachandran K, Kędracka-Krok S, Pabijan J, Lekka M (2022). Discriminating bladder cancer cells through rheological mechanomarkers at cell and spheroid levels. J Biomech.

[176] Vyas V, Solomon M, D’Souza GGM, Huey BD (2019). Nanomechanical analysis of extracellular matrix and cells in multicellular spheroids. Cell Mol Bioeng.

[177] Mahajan V, Beck T, Gregorczyk P, Ruland A, Alberti S, Guck J (2021). Mapping tumor spheroid mechanics in dependence of 3D microenvironment stiffness and degradability by Brillouin microscopy. Cancers (Basel)..

[178] Wullkopf L, West A-KV, Leijnse N, Cox TR, Madsen CD, Oddershede LB (2018). Cancer cells’ ability to mechanically adjust to extracellular matrix stiffness correlates with their invasive potential. Mol Biol Cell.

[179] Tsvirkun D, Revilloud J, Giannetti A, Verdier C (2022). The intriguing role of collagen on the rheology of cancer cell spheroids. J Biomech.

[180] Bae IY, Choi W, Oh SJ, Kim C, Kim S-H (2022). TIMP-1-expressing breast tumor spheroids for the evaluation of drug penetration and efficacy. Bioeng Transl Med.

[181] Goudar VS, Koduri MP, Ta Y-NN, Chen Y, Chu L-A, Lu L-S (2021). Impact of a desmoplastic tumor microenvironment for colon cancer drug sensitivity: a study with 3D chimeric tumor spheroids. ACS Appl Mater Interfaces.

[182] Arora L, Kalia M, Dasgupta S, Singh N, Verma AK, Pal D. Development of a multicellular 3D tumor model to study cellular heterogeneity and plasticity in NSCLC tumor microenvironment. Front Oncol. 2022. 10.3389/fonc.2022.881207 PMC9273950 35837091

[183] Barnett FH, Rosenfeld M, Wood M, Kiosses WB, Usui Y, Marchetti V (2016). Macrophages form functional vascular mimicry channels in vivo. Sci Rep.

[184] Wessels DJ, Pradhan N, Park Y-N, Klepitsch MA, Lusche DF, Daniels KJ (2019). Reciprocal signaling and direct physical interactions between fibroblasts and breast cancer cells in a 3D environment. PLoS ONE.

[185] Bagley JA, Reumann D, Bian S, Lévi-Strauss J, Knoblich JA (2017). Fused cerebral organoids model interactions between brain regions. Nat Methods.

[186] Birey F, Andersen J, Makinson CD, Islam S, Wei W, Huber N (2017). Assembly of functionally integrated human forebrain spheroids. Nature.

[187] Xiang Y, Tanaka Y, Cakir B, Patterson B, Kim K-Y, Sun P (2019). hESC-derived thalamic organoids form reciprocal projections when fused with cortical organoids. Cell Stem Cell.

[188] Sloan SA, Andersen J, Pașca AM, Birey F, Pașca SP (2018). Generation and assembly of human brain region-specific three-dimensional cultures. Nat Protoc.

[189] Nam D, Park MR, Lee H, Bae SC, Gerovska D, Araúzo-Bravo MJ (2022). Induced endothelial cell-integrated liver assembloids promote hepatic maturation and therapeutic effect on cholestatic liver fibrosis. Cells.

[190] Marton RM, Pașca SP (2020). Organoid and assembloid technologies for investigating cellular crosstalk in human brain development and disease. Trends Cell Biol.

[191] Hong S, Song JM (2022). 3D bioprinted drug-resistant breast cancer spheroids for quantitative in situ evaluation of drug resistance. Acta Biomater.

[192] Zhao L, Xiu J, Liu Y, Zhang T, Pan W, Zheng X (2019). A 3D printed hanging drop dripper for tumor spheroids analysis without recovery. Sci Rep.

[193] Utama RH, Atapattu L, O’Mahony AP, Fife CM, Baek J, Allard T (2020). A 3D bioprinter specifically designed for the high-throughput production of matrix-embedded multicellular spheroids. iScience.

[194] Dadgar N, Gonzalez-Suarez AM, Fattahi P, Hou X, Weroha JS, Gaspar-Maia A (2020). A microfluidic platform for cultivating ovarian cancer spheroids and testing their responses to chemotherapies. Microsyst Nanoeng.

[195] Lee SI, Choi YY, Kang SG, Kim TH, Choi JW, Kim YJ (2022). 3D multicellular tumor spheroids in a microfluidic droplet system for investigation of drug resistance. Polymers (Basel)..

[196] Rima XY, Zhang J, Nguyen LTH, Rajasuriyar A, Yoon MJ, Chiang C-L (2022). Microfluidic harvesting of breast cancer tumor spheroid-derived extracellular vesicles from immobilized microgels for single-vesicle analysis. Lab Chip.

[197] Amereh M, Edwards R, Akbari M, Nadler B (2021). In-silico modeling of tumor spheroid formation and growth. Micromachines (Basel)..

[198] Hazan RB, Qiao RUI, Keren R, Badano I, Suyama K (2004). Cadherin switch in tumor progression. Ann N Y Acad Sci.

[199] Gloushankova NA, Zhitnyak IY, Rubtsova SN (2018). Role of epithelial-mesenchymal transition in tumor progression. Biochem Mosc.

[200] Wang B, Tan Z, Guan F (2019). Tumor-derived exosomes mediate the instability of cadherins and promote tumor progression. Int J Mol Sci.

[201] Aiello NM, Kang Y (2019). Context-dependent EMT programs in cancer metastasis. J Exp Med.

[202] Singh S, Chakrabarti R (2019). Consequences of EMT-driven changes in the immune microenvironment of breast cancer and therapeutic response of cancer cells. J Clin Med.

[203] Hanson EM, Clements VK, Sinha P, Ilkovitch D, Ostrand-Rosenberg S (2009). Myeloid-derived suppressor cells down-regulate L-selectin expression on CD4+ and CD8+ T cells1. J Immunol.

[204] Lafouresse F, Bellard E, Laurent C, Moussion C, Fournié J-J, Ysebaert L (2015). L-selectin controls trafficking of chronic lymphocytic leukemia cells in lymph node high endothelial venules in vivo. Blood.

[205] Fabricius H-Å, Starzonek S, Lange T. The role of platelet cell surface P-selectin for the direct platelet-tumor cell contact during metastasis formation in human tumors. Front Oncol. 2021. 10.3389/fonc.2021.642761 PMC8006306 33791226

[206] Leivonen S-K, Lazaridis K, Decock J, Chantry A, Edwards DR, Kähäri V-M (2013). TGF-β-elicited induction of tissue inhibitor of metalloproteinases (TIMP)-3 expression in fibroblasts involves complex interplay between Smad3, p38α, and ERK1/2. PLoS ONE.

[207] Kim E-S, Kim M-S, Moon A (2004). TGF-β-induced upregulation of MMP-2 and MMP-9 depends on p38 MAPK, but not ERK signaling in MCF10A human breast epithelial cells. Int J Oncol.

[208] Lungu G, Covaleda L, Mendes O, Martini-Stoica H, Stoica G (2008). FGF-1-induced matrix metalloproteinase-9 expression in breast cancer cells is mediated by increased activities of NF-κB and activating protein-1. Mol Carcinog.

[209] Gonzalez DM, Medici D (2014). Signaling mechanisms of the epithelial-mesenchymal transition. Sci Signaling..

[210] Wendt MK, Smith JA, Schiemann WP (2010). Transforming growth factor-β-induced epithelial–mesenchymal transition facilitates epidermal growth factor-dependent breast cancer progression. Oncogene.

[211] Mao X, Xu J, Wang W, Liang C, Hua J, Liu J (2021). Crosstalk between cancer-associated fibroblasts and immune cells in the tumor microenvironment: new findings and future perspectives. Mol Cancer.

[212] Jiang X, Wang J, Deng X, Xiong F, Zhang S, Gong Z (2020). The role of microenvironment in tumor angiogenesis. J Exp Clin Cancer Res.

[213] Shan Y, You B, Shi S, Shi W, Zhang Z, Zhang Q (2018). Hypoxia-induced matrix metalloproteinase-13 expression in exosomes from nasopharyngeal carcinoma enhances metastases. Cell Death Dis.

[214] Hofmann UB, Westphal JR, Waas ET, Zendman AJ, Cornelissen IM, Ruiter DJ (1999). Matrix metalloproteinases in human melanoma cell lines and xenografts: increased expression of activated matrix metalloproteinase-2 (MMP-2) correlates with melanoma progression. Br J Cancer.

[215] Brooks PC, Strömblad S, Sanders LC, von Schalscha TL, Aimes RT, Stetler-Stevenson WG (1996). Localization of matrix metalloproteinase MMP-2 to the surface of invasive cells by interaction with integrin alpha v beta 3. Cell.

[216] Levental KR, Yu H, Kass L, Lakins JN, Egeblad M, Erler JT (2009). Matrix crosslinking forces tumor progression by enhancing integrin signaling. Cell.

[217] Jodele S, Blavier L, Yoon JM, DeClerck YA (2006). Modifying the soil to affect the seed: role of stromal-derived matrix metalloproteinases in cancer progression. Cancer Metastasis Rev.

[218] Su C, Li J, Zhang L, Wang H, Wang F, Tao Y, et al. The biological functions and clinical applications of integrins in cancers. Front Pharmacol. 2020. 10.3389/fphar.2020.579068 PMC7522798 33041823

[219] Sternlicht MD, Werb Z (2001). How matrix metalloproteinases regulate cell behavior. Annu Rev Cell Dev Biol.

[220] Paszek MJ, Zahir N, Johnson KR, Lakins JN, Rozenberg GI, Gefen A (2005). Tensional homeostasis and the malignant phenotype. Cancer Cell.

